# Soil Layers Matter: Vertical Stratification of Root-Associated Fungal Assemblages in Temperate Forests Reveals Differences in Habitat Colonization

**DOI:** 10.3390/microorganisms9102131

**Published:** 2021-10-11

**Authors:** Anis Mahmud Khokon, Dominik Schneider, Rolf Daniel, Andrea Polle

**Affiliations:** 1 Department of Forest Botany and Tree Physiology, University of Göttingen, 37077 Göttingen, Germany; anis.khokon@gwdg.de; 2 Genomic and Applied Microbiology and Göttingen Genomics Laboratory, Institute of Microbiology and Genetics, University of Göttingen, 37077 Göttingen, Germany; dschnei1@gwdg.de (D.S.); rdaniel@gwdg.de (R.D.)

**Keywords:** root-associated fungi, vertical soil layers, high throughput sequencing, beta diversity, indicator fungal order, indicator fungal taxa, temperate forests

## Abstract

Ectomycorrhizal and saprotrophic fungi play pivotal roles in ecosystem functioning. Here, we studied the vertical differentiation of root-associated fungi (RAF) in temperate forests. We analysed RAF assemblages in the organic and mineral soil from 150 experimental forest plots across three biogeographic regions spanning a distance of about 800 km. Saprotrophic RAF showed the highest richness in organic and symbiotrophic RAF in mineral soil. Symbiotrophic RAF exhibited higher relative abundances than saprotrophic fungi in both soil layers. Beta-diversity of RAF was mainly due to turnover between organic and mineral soil and showed regional differences for symbiotrophic and saprotrophic fungi. Regional differences were also found for different phylogenetic levels, i.e., fungal orders and indicator species in the organic and mineral soil, supporting that habitat conditions strongly influence differentiation of RAF assemblages. Important exceptions were fungal orders that occurred irrespective of the habitat conditions in distinct soil layers across the biogeographic gradient: Russulales and Cantharellales (ectomycorrhizal fungi) were enriched in RAF assemblages in mineral soil, whereas saprotrophic Polyporales and Sordariales and ectomycorrhizal Boletales were enriched in RAF assemblages in the organic layer. These results underpin a phylogenetic signature for niche partitioning at the rank of fungal orders and suggest that RAF assembly entails two strategies encompassing flexible and territorial habitat colonization by different fungal taxa.

## 1. Introduction

Fungi are a remarkably diverse group of organisms on Earth, playing pivotal roles in terrestrial biogeochemical cycles [1,2]. In temperate forests, saprotrophic, symbiotrophic, and pathotrophic fungi have distinct tasks in carbon and nutrient cycling [3]. Saprotrophic fungi are mainly responsible for decomposing plant litter in the forest floor and for maintaining carbon cycling, whereas symbiotrophic fungi such as mycorrhizal fungi colonize roots and mine the soil for mineral nutrients, which they deliver to the plant in exchange for photosynthetically derived carbohydrates [3]. Pathogenic fungi cause diseases and thereby may eventually structure the composition of the vegetation [4]. A small change in these microbial structures can significantly impact matter fluxes and ecosystem functioning [5].

Because of their eminent roles in nutrient cycling in forests, the assembly processes of belowground fungal communities have received increasing attention. In general, fungal assemblages are not occurring at random [6,7]. The composition of soil fungal communities is mainly driven by habitat conditions [8,9,10]. Environmental factors with strong effects on the fungal community composition in soil of temperate forests include temperature, precipitation, soil properties, vegetation, etc., [8,11,12,13,14]. 

In addition to forest soil, roots are an important habitat for belowground fungi [15]. The fungi colonize the rhizoplane and grow as saprotrophs or interact with living tissue as endophytes, pathogens or symbionts [15]. Based on their habitat, these fungi have been defined as root-associated fungi (RAF) [16] and are considered as critical components of the plant microbiome [15]. Like soil fungal assemblages, the RAF composition in temperate forests is shaped by various biotic and abiotic environmental conditions including tree species, soil pH, soil moisture, availability of mineral nitrogen and phosphorus and the soil C/N ratio [17,18,19,20,21]. However, along large-scale environmental gradients these factors have lower impact on the composition of RAF than on the composition of fungi in soil [17]. Therefore, it was suggested that the RAF community composition is influenced by additional factors than the soil residing fungi [17], for instance by root properties, especially root N contents [18,20]. 

Soil depth is a further important factor structuring fungal communities [19,22,23,24,25,26]. The forest floor consisting of decaying organic material is characterized by high organic carbon (C) and N contents and a wider C/N ratio, whereas the mineral top soil contains narrower C/N ratios and generally decreasing availabilities of nutrient resources [27,28]. The transition from the organic layer to mineral top soil often shows a sharp border with an associated drastic shift in soil chemistry [27,28]. The structuring gradient of soil layers has mainly been studied for soil fungi, showing that saprotrophic fungi were more abundant in the uppermost soil layers composed of fresh litter and decomposing organic material, whereas mycorrhizal fungi dominated deeper in the mineral soil [19,22,29,30]. The influence of soil horizons on fungal composition was even stronger than that of other environmental factors [19,26]. Fine scale analyses of vertical distribution of fungi have also been conducted in different soil depth and showed vertical niche partitioning of ectomycorrhizal fungi on roots [31], fungal hyphae [32], and both [33]. Despite the huge impact of soil horizons on structuring fungal assemblages, studies on the stratification of RAF in organic and mineral soil are scarce and entirely lacking for large-scale biogeographic regions. 

The goal of this study was to enhance our understanding of fungal niche partitioning by studying commonalities and differences of the composition of RAF communities in the organic layer and mineral top soil across different biogeographic regions. For this purpose, we used 150 well-established plots of a large-scale infrastructure for biodiversity studies (Biodiversity Exploratories: https://www.biodiversity-exploratories.de/en/, accessed on 10 May 2017) in typical European forests mainly composed of beech (*Fagus sylvatica*) and conifers (*Picea abies*, *Pinus sylvestris*, [34]). The Biodiversity Exploratories are located in the northeast, the middle and the southwest of Germany, encompassing areas of about 450 to 1300 km^2^. The northeast is characterized by drier and warmer climate and sandy soils, whereas the middle and the southwest have lower temperatures, higher soil moisture and silty or loamy soils [34,35,36]. We used this set-up to investigate the fungal assemblages of roots in organic and mineral soil. We know from our previous studies that the tree roots in our forest plots are massively colonized by ectomycorrhizal fungi [37] but how different fungal guilds on roots are affected by soil strata is unknown. Here, we hypothesized that (i) the species richness and relative abundance of symbiotrophic fungi is higher than that of saprotrophic fungi in RAF assemblages, irrespective of the soil layer. We further hypothesized that (ii) the taxonomic community composition of symbiotrophic and saprotrophic fungi differs significantly between organic layer and mineral soil and shows lower turnover for symbiotrophic than for saprotrophic fungi between the soil layers. We hypothesized that (iii) RAF patterns indicate response traits either to soil strata or to regional habitat conditions. To test the third hypothesis, we compared the responses of phylogenetically related taxa (at the rank of fungal orders) to mineral and organic soil in different biogeographic regions. Moreover, we expected to discover root-associated indicator species for distinct soil layers and for different biogeographic regions representing territorial or flexible behaviour. 

## 2. Materials and Methods

### 2.1. Study Sites Characteristics 

This study was conducted in 150 experimental forest plots in the Biodiversity Exploratories (https://www.biodiversity-exploratories.de/en/, accessed on 10 May 2017, [34]). The Biodiversity Exploratories are located in three geographic regions: Schorfheide-Chorin (SCH) in the northeast, Hainich-Dün (HAI) in the center and Schwäbische Alb (ALB) in the southwest region of Germany. The plots locations are indicated in Supplementary Materials (Table S1). The soil types vary among the regions, with silty soils in ALB, loamy soils in HAI and sandy soils in SCH [36] (Table 1). The plots are located in 53- to 141-year-old forest stands mainly composed of Fagaceae (*Fagus sylvatica* or *Quercus* sp.) and Pinaceae (*Picea abies* and *Pinus sylvestris*) [34,38]. Additional soil and climatic characteristics have been compiled in Table 1.

### 2.2. Root and Soil Sampling from Organic and Mineral Soil Layers 

In each region, roots and soil were sampled in 50 experimental plots [34] in May 2017. In each plot, two transects of 40 m length from north to south and from east to west were established and soil was collected at a distance of 4.5, 10.5, 16.5, 22.5, 28.5, 34.5 and 40.5 m along the transects as described previously [20]. At each sampling point, organic layer (Oe horizon = moderately decomposed organic material with a proportion of visible plants residues between 16 and 66% and Oa horizon = strongly decomposed organic materials with less than 16% visible plant residues) and mineral top soil (to a depth of 10 cm) were sampled separately, resulting in 14 samples per soil layer. In each plot, the samples per layer were combined to one sample of the organic and one sample of the mineral soil. Subsequently, the soil was sieved. Fine roots (<2 mm in diameter) were collected and washed in tap water. Approximately 1 g of fine roots were frozen in liquid nitrogen in the field and stored at −80 °C. The remaining roots were collected, dried (60 °C) and the biomass was recorded (Supplementary Materials Table S1). Aliquots of soil samples (approximately 300 g) were stored at 4 °C.

### 2.3. Determination of Soil Chemical Properties

Carbon and nitrogen: soil samples were dried at 40 °C for two weeks and ground to a homogenous fine powder with a ball mill (Retsch, Type MM400, Haan, Germany). Aliquots of dry soil powder (10 mg organic soil, 30 mg mineral soil) were weighed in in 4 mm × 6 mm tin capsules (IVA Analysentechnik, Meerbusch, Germany) using a super-micro balance (S4, Sartorius, Goettingen, Germany). Total soil carbon (C) and nitrogen (N) were measured by dry combustion in a CN analyzer “Vario Max” (Elementar Analysensysteme GmbH, Hanau, Germany). Acetanilide (71.09% C, 10.36% N) was used as the standard.

Phosphorus and basic cations: potentially plant available phosphorus (P_sol_) was extracted according to the method of Bray and Kurtz [39]. Approximately 100 mg of dry soil powder was mixed with 150 mL of Bray extraction solution (0.03 N NH_4_F, 0.025 N HCl). The suspension was shaken slowly for 60 min on a rotary shaker and subsequently filtered through phosphate-free paper filters (MN 280 1/4 125 mm, Macherey–Nagel, Düren, Germany). P_sol_ was measured in the filtrate by inductively coupled plasma–optical emission mass spectroscopy (ICP-OES) (iCAP 7000 Series ICP–OES, Thermo Fisher Scientific, Dreieich, Germany). To determine the potassium (K), calcium (Ca) and magnesium (Mg) contents, approximately 40 to 50 mg soil powder was extracted in 25 mL of 65% HNO_3_ (Merck, Darmstadt, Germany) for 12 h at 160 °C [40]. The suspension was filtered (filter papers MN 640 w, width 90 mm, Macherey–Nagel, Düren, Germany) and the filtrate used for element determination by ICP-OES (iCAP 7000 Series, Thermo Fischer Scientific). The calibration was performed with standards of 1 g L^−1^ (Einzelstandards, Bernd Kraft, Duisburg, Germany). The cations per sample (mmol g^−1^ dry soil) were added and the sum of cation was used in further analyses.

### 2.4. DNA Extraction and Polymerase Chain Reaction

Frozen fine roots that had been stored at −80 °C were milled with a Retsch ball mill (Type MM400, Retsch GmbH, Haan, Germany) at a frequency of 30 s^−1^ for 3 min in liquid nitrogen. The genomic DNA was extracted from the frozen root powder with the innuPREP Plant DNA Kit (Analytik Jena AG, Jena, Germany), following the manufacturer’s instructions. We used the internal transcribed spacer (ITS) region 2 for fungal identification as recommended by Horton and Bruns [41]. To amplify the fungal ribosomal ITS2 region in roots, we used ITS3_KYO2 [42] as the forward primer (GATGAAGAACGYAGYRAA) and ITS4 [43] as the reverse primer (TCCTCCGCTTATTGATATGC). The primers contained adapter sequences for MiSeq sequencing (Illumina Inc., San Diego, CA, USA). The polymerase chain reactions (PCR) were conducted as described elsewhere [20]. The size of the PCR products was determined after electrophoresis in 2% agarose gels (Biozym Scientific GmbH, Hessisch Oldendorf, Germany) with a DNA standard (Thermo Scientific^TM^ GeneRuler^TM^ 1kb DNA Ladder, Life Technologies GmbH, Darmstadt, Germany). The PCR products were purified with a magnetic bead-based Magsi-NGS*^PREP^* kit (Steinbrenner Laborsysteme GmbH, Wiesenbach, Germany). PCR products were stained using GelRed (0.01 μL mL^−1^, GelRed^TM^ Nucleic Acid, Biotium Inc., VWR International GmbH, Darmstadt, Germany). The PCR products were visualized with an FLA-5100 Fluorescence Laser Scanner (Raytest GmbH, Straubenhardt, Germany) and an Aida Image Analyser v. 4.27 (Raytest GmbH). Purified PCR products were quantified using a Qubit dsDNA HS assay Kit in a Qubit 3.0 Fluorometer (Thermo Fischer Scientific, Dreieich, Germany). Amplicon sequencing was conducted on the Illumina MiSeq platform using the MiSeq Reagent Kit v3 (Illumina Inc., San Diego, CA, USA) at the Göttingen Genomics Laboratory, Germany.

### 2.5. Bioinformatics Processing and Analyses

The raw paired-end reads were quality-filtered with fastp v0.20.0 using the following settings: phredscore of threshold 20, overlapping base pair (bp) correction, sliding window size of 4 and a minimum length of 50 bp [44]. The resulting paired-end ITS sequences were merged (*--fastq_mergepairs*) using PEAR v0.9.11 [45]. Reverse and forward primers were clipped (*-g for_primer –a rev_primer-trim-n*) by employing cutadapt v2.5 with default settings [46]. To generate amplicon sequence variants (ASVs) [47], high quality ITS2 sequence reads were processed with VSEARCH v2.14.1 [48], which included the following steps in order: size sorting and filter (*-sortbylength*) that discarded ITS2 sequences shorter than 140 bp, dereplication (*-derep_fulllength-sizeout*), and denoising (*-cluster_unoise-minsize* 8) [49]. Subsequently, operational taxonomic units (OTUs) were generated by clustering the ASVs at 97% sequence identity (*-sortbysize* & *-cluster_size*). All quality-filtered merged reads were mapped to chimera-free OTUs, and an OTU abundance table was created using VSEARCH (*-usearch_global-id 0.97*). The taxonomic classification of the OTUs was extracted from the UNITE database v8.2 [50] by employing BLASTn, version 2.9.0+. Taxonomic information was added to the OTU abundance table with BIOM tools [51]. All unidentified fungal ASVs were searched (BLASTn) [52] against the nt database (17 January 2020) to remove non-fungal OTUs. Only OTUs with a fungal classification were kept in the OTU table.

Applying this pipeline, we obtained a total number of 2.6 and 4.0 million sequences for the root samples from the organic and mineral soil layers, respectively. The OTU table was rarefied to 3890 reads per sample (minimum number of counts in a sample) by employing the function *amp_subset_samples()* from the ‘ampvis2′ package implemented in R [53]. Rarefaction resulted in a total number of 2046 and 2147 distinct OTUs for roots from the organic and mineral soil, respectively. Fungal OTUs were functionally annotated as symbiotroph (SYM), saprotroph (SAP) and pathotroph (PAT) using the program ‘FunGuild’ [54].

### 2.6. Data Processing and Statistical Analysis

The statistical analyses were performed in R version 4.0.3 [55]. Data distribution and homogeneity of the variances were inspected visually using histograms and residual plots. Data were logarithmically transformed to meet the criteria of normal distribution and homogeneity of variances, where necessary. Generalized linear models (Poisson regression, chi-square test) were used with the function *glm()* from ‘lme4′ package to investigate datasets with count data (fungal species richness, sequence reads) [56]. Linear models were used to investigate the datasets with continuous data (C, N, C/N, P_sol_, sum of cations) with the function *lm()* from ‘lme4′ package [56]. Differences among variables were calculated using the *Anova()* function from the ‘car’ package. Pairwise differences between different groups were compared with a *post hoc test* (HSD Tukey’s honestly significant difference) using the function *glht()* from the ‘multcomp’ package [57]. Differences were considered to be significant when *p* ≤ 0.05.

We investigated the dissimilarities of RAF communities by non-metric multidimensional scaling (NMDS) ordination. We performed NMDS analyses with the function *metaMDS()* in ‘vegan’ package [58] with three dimensions, 100 iterations and Bray-Curtis as the dissimilarity measure. Effects of the soil layers on fungal community composition were tested using Analysis of Similarities (ANOSIM with 999 permutation steps) with the function *anosim()* as implemented in ‘vegan’ package.

We used “generalized additive models for location scale and shape” (GAMLSS) with a zero-inflated beta (BEZI) family (GAMLSS-BEZI) from the ‘metamicrobiomeR’ package [59] to compare the relative abundance of the fungal orders between organic and mineral soil layers. Fungal orders were filtered applying the threshold of >0.5% of the mean sequence abundances using the function *taxa.filter()* to exclude low-abundant fungal orders from further analysis. The relative abundances of the root fungal orders from the organic and mineral soil layers were compared using the function *taxa.compare()*. The *p*-values were adjusted using the default method ‘False Discovery Rate’ (FDR). The analyses were conducted for each of the three study regions separately and then a meta-analysis was performed using the function *meta.taxa()* to estimate the overall effects across the three regions with “region” as random factor. In the meta-analysis, the model pooled adjusted estimates and standard errors with inverse variance weighting and the DerSimonian-Laird estimator from each region to determine the overall effects across regions. Heatmap and forest plot were generated using the function *meta.niceplot()*. The heatmaps show log-odds ratio (log(OR)), i.e., effect sizes indicating enrichment or depletion of fungal orders between organic and mineral soil layer. The fungal orders were grouped according to their inferred ecological functions as SYM or SAP. A fungal order was regarded as SYM, when >95% of sequences per order were annotated as SYM or as SAP when >95% of the sequences of the given orders were assigned as SAP. If a fungal order could not be assigned to SYM or SAP, it was classified as multiple functional mode such as SAP+PAT, SAP+SYM, etc.

To determine the β-diversity (Sørensen dissimilarity), the abundance-based OTU data matrices were transformed into presence-absence (1 or 0) matrices with the function *beta.temp()* from the ‘betapart’ package [60]. Total β-diversity was further partitioned into species turnover, i.e., replacement of taxa between organic and mineral soil layer and nestedness, i.e., loss of taxa between organic and mineral soil layer using ‘betapart’. Pairwise differences of each response variable (e.g., overall beta diversity, nestedness and turnover components) and each fungal group (e.g., all fungi, SYM and SAP) were compared separately among the regions, using a *post hoc* test (HSD Tukey’s honestly significant difference). Further, *paired rank sum test* was used to compare the different fungal functional group in each region.

Bipartite networks were built to evaluate associations of fungal taxa (OTUs) with organic and mineral soil layers, using the ‘bipartite’ package [61]. We included OTUs with relative abundances >0.1% of the fungal sequences in this analysis. The *plotweb()* function was used to visualize bipartite network plots. To determine fungal indicator taxa for roots in the organic and mineral soil layer, we used the *multipatt()* function from the ‘indicspecies’ package [62]. Further, the significance difference of the enrichment of the SYM and SAP taxa between the soil layers were tested using the function *fischer.test()*. Data were visualized using ‘ggplot2’ package [63] in R.

## 3. Results

### 3.1. Differences in Soil Chemistry among Different Biogeographic Regions Are Larger in the Mineral Topsoil Than in the Organic Layer

Across the three regions, which spanned a geographic distance of about >800 km, the organic soil was characterized by higher concentrations of carbon, nitrogen, basic cations and potentially soluble P than the mineral top soil (Table 2). In the organic layer, C/N ratios exhibited relatively stable values of approximately 22 across the three regions (Table 2). In the mineral soil, C/N ratios varied among the regions and were approximately 30% lower in ALB and HAI than in SCH (Table 2). Mean P_sol_ concentrations varied among the regions, about 1.5-fold in the organic and 2-fold in the mineral soil (Table 2). The basic cation concentrations varied among the regions, about 3.6-fold in the organic and 7.7-fold in the mineral soil (Table 2). Overall, regional differences were stronger in the mineral top soil than in the organic layer (Table 2).

### 3.2. Strong Taxonomic Differentiation of Root-Associated Fungal Assemblages between Organic and Mineral Topsoil 

Our analyses of RAF in 300 root samples from two different soil layers in three biogeographic regions resulted in a rarefied data set containing 1.2 Mio fungal sequences, which clustered into 2537 different fungal OTUs. The mean OTU richness per plot ranged from 158 to 188 for the roots in the organic layer and from 106 to 157 in roots from the mineral top soil (Table S2). For both soil strata and all fungal guilds, lowest fungal richness was found in SCH compared to HAI or ALB (Table S2).

**Figure 1:**
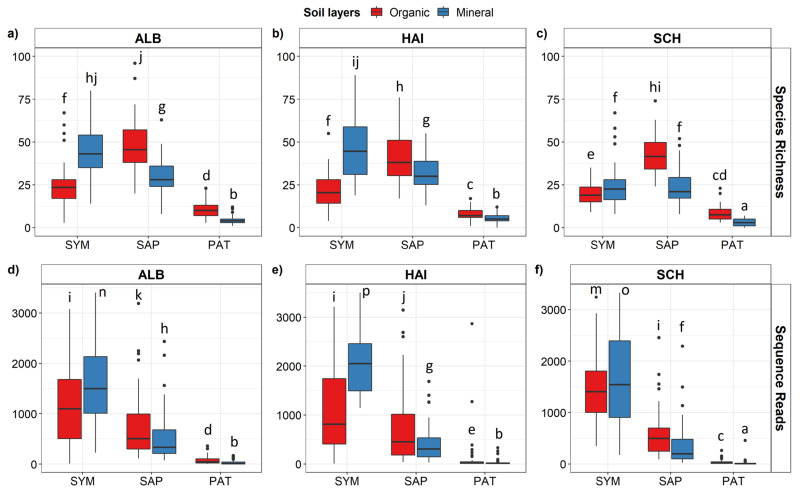
Species richness (**a–c**) and abundance (**d–f**) of root-associated fungi in the organic layer and mineral topsoil in three biogeographic regions. ALB = Schwäbische Alb, HAI = Hainich-Dün, SCH = Schorfheide-Chorin. SYM = Symbiotroph, SAP = Saprotroph and PAT = Pathotroph. Abundances are indicated as the number of sequences with a total number of 3890 sequences per sample. Data indicate means ± SE (*n* = 50). Generalized linear model with Poisson regression and chi-square test was used to compare the fungal groups between soil layers. Pairwise differences of the functional groups of root-associated fungi between organic layer and mineral soil were compared using a *post hoc test* (HSD Tukey’s honestly significant difference). Different letters indicate significant differences of the means at *p* ≤ 0.05.

SAP and PAT fungi on the roots exhibited higher OTU richness in the organic than in the mineral layer (Figure 1a–c), while the richness of the SYM fungi was higher in the mineral than in the organic soil (Figure 1a–c). Similarly to OTU richness, the number of SYM sequences was higher on roots in the mineral than in the organic layer, while SAP and PAT sequences were enriched on roots from the organic compared to the mineral layer (Figure 1d–f). Overall, OTU richness of PAT and their relative abundances (3% of the total number of sequences) were low compared to SYM (54%) or SAP (31%) (Figure 1).

**Figure 2:**
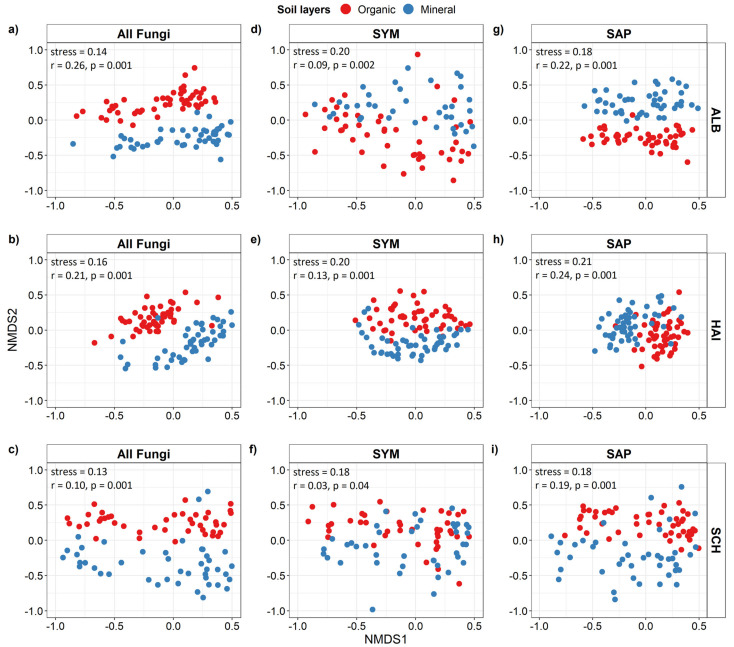
Non-metric multidimensional scaling (NMDS) of root-associated fungal community composition of all (all OTUs, (**a–c**)), symbiotrophic (SYM, (**d–f**)) and saprotrophic (SAP, (**g–i**)) fungi in three biogeographic regions. ALB = Schwäbische Alb, HAI = Hainich-Dün and SCH = Schorfheide-Chorin (*n* = 50 per region and soil stratum). Dissimilarities were calculated with the Bray-Curtis distance measure. Red = organic layer, blue = mineral soil. Significant differences of root-associated fungal community composition between soil layers were tested with analysis of similarities (ANOSIM) using 999 permutations and the ‘Bray Curtis’ distance method.

In each region, the RAF assemblage in the organic layer was clearly separated from that in the mineral soil (Figure 2a–c), showing dissimilarities of fungal communities on roots in different soil layers. These separations were significant (ANOSIM, *p* and *r* values in Figure 2a–c). Significant separations between the organic and mineral soil were also found for SYM and SAP, the main functional fungal groups colonizing roots (SYM: Figure 2d–f, SAP: Figure 2g–i). The goodness-of-fit (*r* values in Figure 2 d–i) was greater for SAP than for SYM assemblages, indicating stronger dissimilarities for SAP than for SYM assemblages on roots in different soil layer. Overall, our data show a strong vertical differentiation of the taxonomic and functional composition of the RAF between organic and mineral soil layers (Figure 2).

### 3.3. β-Diversity of Root-Associated Fungal Taxa between Organic and Mineral Soil Shows Regional Differences

**Figure 3:**
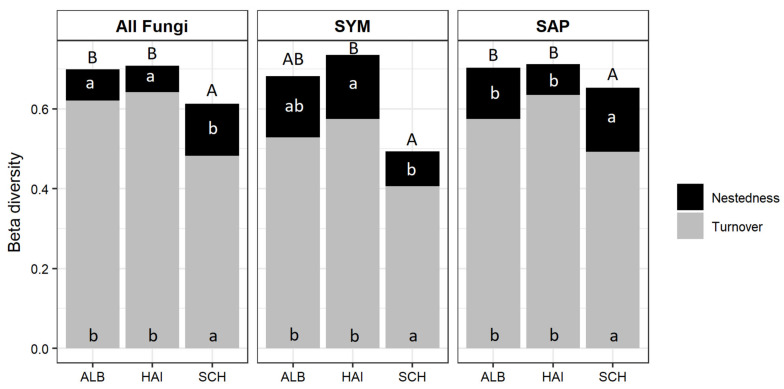
Beta-diversity of the root-associated fungal community composition of all (all OTUs), symbiotroph (SYM), and saprotroph (SAP) fungi between the organic layer and mineral soil. Stacked bars represent overall β-diversity (β_SOR_) for the three regions. ALB = Schwäbische Alb, HAI = Hainich-Dün and SCH = Schorfheide-Chorin. Grey sections of the bars represent the mean fungal turnover (β_SIM_), and black sections represent the mean fungal nestedness (β_SNE_). Pairwise differences of each variable were compared separately among the regions using a *post hoc* test (HSD Tukey’s honestly significant difference). Different letters represent significant differences of the means at *p* ≤ 0.05. Capital letters refer to significant differences for the overall beta diversity and small letters for the turnover and nestedness components of the beta diversity.

We found significant differences for the β-diversity of the fungal community composition among the three regions, which were caused by lower β-diversity of RAF in SCH than in the other two regions (Figure 3; Table S3). We partitioned total β-diversity into the turnover component, i.e., replacement of taxa between organic and mineral soil layers, and nestedness, i.e., loss of taxa between organic layer and mineral soil. We found significant differences in RAF turnover among the regions, irrespective of the fungal group analysed with higher turnover in ALB and HAI than in SCH (Figure 3; Table S3). Nestedness of all fungi was higher in SCH than ALB or HAI (Figure 3). The enhanced higher nestedness in SCH was caused by enhanced nestedness of SAP since the SYM nestedness was unaffected (Figure 3). In general, the extent of the turnover accounted for approximately 80% of the β-diversity of RAF assemblages (Figure 3). We further compared the turnover and nestedness components of SYM and SAP fungi in each region separately. We found significant differences of SYM and SAP fungal turnover and nestedness in HAI (turnover: test statistics = 2.10, *p* = 0.04; nestedness: test statistics = 3.21, *p* = 0.001) and SCH (turnover: test statistics = 3.21, *p* = 0.001; nestedness: test statistics = 3.20, *p* = 0.001) but not in ALB (turnover: test statistics = 1.65, *p* = 0.10; nestedness: test statistics = 1.01, *p* = 0.31).

### 3.4. Phylogenetically Related Fungal Groups Show Divergent Responses to Organic Layer and Mineral Soil

**Figure 4:**
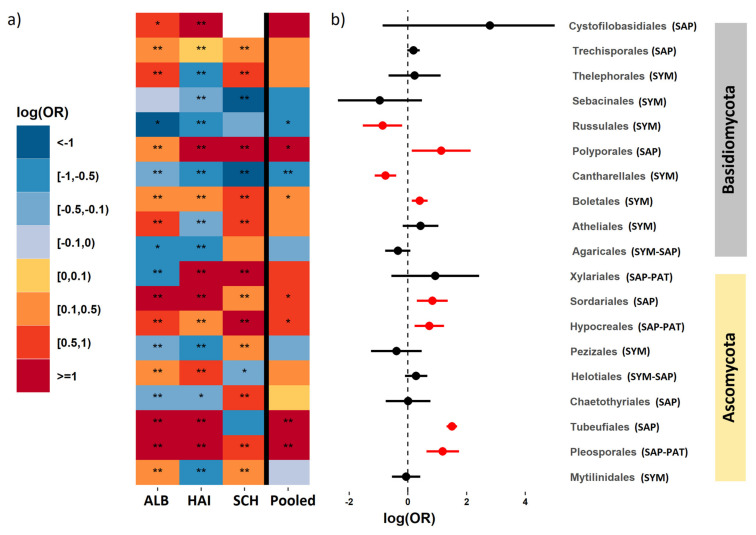
Effect sizes of the changes of root-associated fungal orders between organic to mineral soil. The most abundant fungal orders with >0.5% of the total number of sequences were included. Data are displayed as a heatmap of the log-odds ratio (log(OR)), which indicates the abundance in organic layer relative to that in the mineral soil. Data show the results for three regions (**a**) and the forest plot of pooled estimates across all three regions with 95% confidence intervals (**b**). ALB = Schwäbische Alb, HAI = Hainich-Dün, SCH = Schorfheide-Chorin. Red and blue colours indicate the enrichment of the root-associated fungal order in the organic layer and mineral soil, respectively. The order of Cystofilobasidiales was not present in the SCH region. Significant differences at *p* < 0.05 are denoted with * and at *p* < 0.001 with **. Significant pooled log-odds ratio (OR) estimates with *p* < 0.05 are shown in red colour in the forest plot (**b**).

We classified OTUs according to fungal orders and selected 19 orders, each representing at least 0.5 % or more of the total number of sequences (Table S4). The selected orders accounted together for 85 % of the total number of sequences (Figure S1). Eight of the 19 fungal orders were assigned as SYM, six as SAP, two as SYM+SAP and three as SAP+PAT guilds (Table S5). In most regions, we found significant differences in the relative abundance of the selected RAF orders between organic and mineral soil layers (Figure 4a). However, the responses did not always show the same direction in different regions (Figure 4a), thus, resulting only in eight significantly affected orders across all study regions (Figure 4b). The orders of Polyporales and Sordariales, which contained only SAP fungi, were enriched on roots from the organic layer compared to those from the mineral soil (Figure 4a,b). Significant enrichment on roots from the organic layer was also observed for the Hypocreales and Pleosporales, orders, which were composed of saprotrophic and pathotrophic fungi (Figure 4). Russulales and Cantharellales, orders containing SYM fungi, were enriched on roots in the mineral soil (Figure 4a,b). In contrast to Russulales and Cantharellales, Boletales, also a SYM order, was enriched on the roots from the organic layer (Figure 4a,b).

Heterogeneous enrichment patterns between different soil layers were observed for SYM fungal orders (Thelephorales, Atheliales, Pezizales and Mytilinidales) as well as for SAP fungal orders (Chaetothyriales and Tubeufiales). In most cases, RAF orders in ALB and HAI showed opposing responses compared to SCH (Figure 4a).

### 3.5. Fungal Indicator Taxa in Organic Layer and Mineral Soil

**Figure 5:**
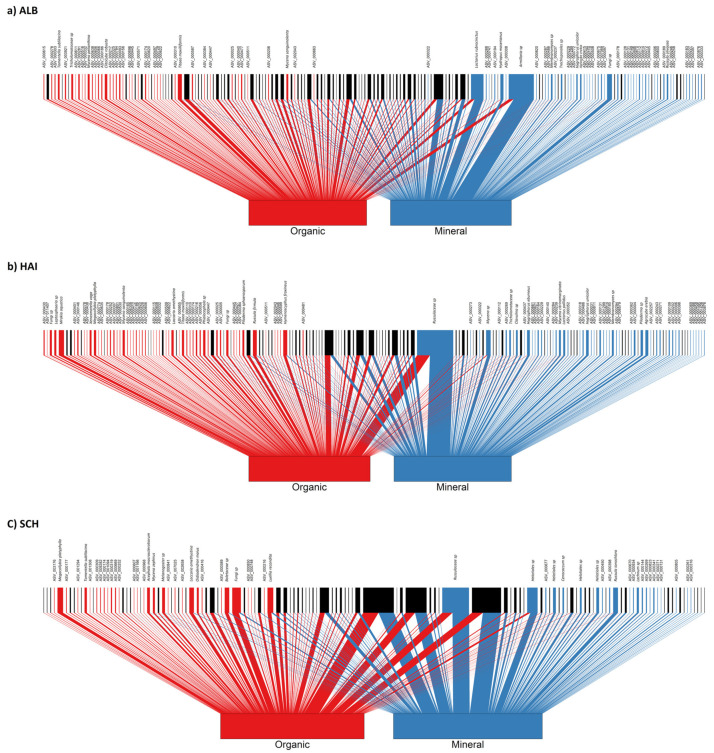
Bipartite network associations of root-associated fungi between organic layer and mineral soil in three biogeographic regions. (**a**) ALB = Schwäbische-Alb, (**b**) HAI = Hainich-Dün and (**c**) SCH = Schorfheide Chorin. Upper nodes refer to fungal OTUs, and lower nodes refer to organic layer (red) and mineral soil (blue). The width of the upper nodes reflects the relative abundance of fungal OTUs from both soil layers. Line widths represent the relative abundances of the fungal OTUs from the respective soil layer. The statistically significant associations of root-associated fungal OTUs are shown in red for the organic layer and in blue for the mineral soil in the upper nodes. Black nodes represent non-significant fungal OTUs associations between the organic and mineral soil layers.

Bipartite network association analysis was performed to determine the degree of habitat preference of distinct taxa (OTU based) in the organic layer and mineral soil (Figure 5).

In ALB, 82 fungal taxa were significantly associated with roots with equal numbers of taxa (41) being significantly associated with the organic and mineral soil (Figure 5a). A similar pattern was observed for HAI (OTUs: organic = 44 and mineral = 43, Figure 5b) but not in the SCH region (OTUs: organic = 31 and mineral = 18, Figure 5c). Across the regions, we found that 30 of 39 SAP indicator species (77%) were present on roots from the organic layer and that 52 of 63 SYM indicator species (81%) were present on roots in the mineral soil layer (Table S6). The enrichment of SYM in the mineral and of SAP indicator taxa in the organic layer was significant (*p* < 0.001, *Fisher’s exact test*).

Only three taxa were shared indicator species in the three study regions: *Calycellina fagina* (Helotiales, SAP) and *Laccaria amethystina* (Agaricales, SYM) in the organic layer and a member of the *Russulaceae* family (ASV_000131, SYM) in the mineral soil (Table S6, Supplementary Materials). Five SAP (*Megacollybia platyphylla, Titaea maxilliformis, Luellia recondita, Apodus* sp., *Cladophialophora* sp.) and two SYM taxa (*Melanogaster* sp., *Tomentella sublilacina*) occurred in two regions as indicator species for roots from the organic layer (Table S6, Supplementary Materials). Seven SYM taxa (*Hygrophorus unicolor*, *Tuber* sp., *Lactarius pallidus*, *Amanita* sp., *Russula foetens*, *Russula* sp., *Glomeraceae* sp.) and three SAP taxa (*Lachnum* sp., *Hydropus moserianus*, *Agrocybe erebia*) occurred in two regions as indicator species for roots in the mineral soil (Table S6, Supplementary Materials). In most cases, HAI and ALB shared indicator species, while SCH rarely shared indicator species with any of the other regions (Table S6, Supplementary Materials).

## 4. Discussion

### 4.1. Stratification of Root-Associated Fungi by Organic Layer and Mineral Topsoil

Here, we characterized the assembly patterns of fungi associated with roots in two major soil strata, the organic layer and the mineral top soil across a large biogeographic scale. In agreement with studies on soil-localized fungi [22,26,64,65,66,67,68,69], we found a clear separation of the RAF communities according to soil layers in each of the three biogeographic areas. In line with targeted analyses of mycorrhizal fungi colonizing the roots [19,29,70,71], we found a clear separation of symbiotrophic as well as of saprotrophic RAF communities by soil strata. Pathogenic fungi were rare (relative abundance of 1.3% to 4.0%), most likely occurring by chance, as suggested previously [18]. Overall, the consistent separation of the fungal groups according to soil strata indicate that soil quality is a dominant factor across a large biogeographic scale.

Saprotrophic fungi usually prefer the organic layer, where they degrade organic material to obtain C and contribute to the decomposition of organic substances in forest soil [72,73]. Mycorrhizal fungi mine the mineral soil for inorganic compounds and rely on host-derived C, and therefore may reside in older litter layers and mineral topsoil [74]. Our results show that soil C, N, C/N ratio, P_sol_ and cations differed strongly between the organic layer and mineral top soil, similar to other studies [19,27,28]. Thus, the decline in soil nutrients from the organic layer to the mineral topsoil would support the well-known shift from saprotrophic to symbiotrophic fungi [19,22,75], if the RAF assemblage was only driven by soil properties. However, in support of our initial hypothesis, the relative abundance of symbiotrophic fungi was higher on roots in both soil strata, most likely because mycorrhizal fungi have direct access to carbohydrates from their host, irrespective of the soil layer and therefore have an advantage over saprotrophic fungi colonizing the rhizoplane. Still, strong influence of soil layers on the composition of the fungal assemblages was evident, as the richness of saprotrophic fungi was higher under nutrient-rich conditions (organic layer), while the richness of symbiotrophic fungi was higher in nutrient-poor conditions (mineral soil). These results are in line with the results on soil fungi in boreal forests [22,75] and arbuscular mycorrhizal fungi in temperate forests [19]. Whether the shifts in the richness are the result of different nutritional acquisition strategies of saprotrophic and symbiotrophic fungi or of antagonistic relationships requires further studies. Future studies should also address the impact of root necromass on RAF diversity.

Since we conducted our study in different biogeographic regions, we were able to detect regional effects on the vertical distribution of RAF. This is a novel aspect shedding light on fungal assemblies. For example, RAF richness and β-diversity were lower in the region with drier, more acidic and nutrient-poor forest soil than in regions with cooler, moist climate and nutrient-rich soil. This can be explained by the classical ecological theory that environmental stress conditions result in reduction of species diversity, selecting species that can tolerate harsh condition [76]. Mycorrhizal fungi are known to be sensitive to elevated temperature and drought [77,78,79,80,81]. Therefore, the climatic differences might have negatively affected the richness of the symbiotrophic fungi in the SCH region compared to ALB or HAI, where the soils have a higher water-holding capacity [36]. In addition, more fertile soil conditions, as present in ALB and HAI, may have favoured higher mycorrhizal diversity, in line with previous studies [12,18,20,82].

At the plot level, we observed a low nestedness and high turnover of RAF between the soil strata but the turnover of symbiotrophic fungi was lower than that of saprotrophic fungi. This result was expected (hypothesis ii) since mycorrhizal fungi colonizing roots are directly influenced by roots of their host. Host effects are known from studies showing strong impact of vegetation [12,14,37], tree identity [70,83,84,85] and root chemistry [20,86]. Host effects may stabilize mycorrhizal patterns at larger geographic scales [17]. Here, we found lower turnover of symbiotrophic than of saprotrophic fungi within the RAF assemblies. Therefore, the present results indicate that root traits may drive symbiotrophic differently from saprotrophic fungi by stabilizing the communities. However, it should be noted that the differences between the turnover of saprotrophic and symbiotrophic fungi were relatively small compared to the overall turnover, underpinning strong abiotic habitat impact on the RAF.

### 4.2. Indicator Taxa and Phylogenetic Groups Uncover Different Strategies of Root-Associated Fungi

An important goal of this study was to test the hypothesis that RAF patterns indicate response traits either to soil strata or to regional habitat conditions. We chose a novel approach by grouping the fungal taxa according to their phylogenetic rank at the order level and measuring the effect size imposed by soil strata on in the fungal abundance in different regions and across all regions. Phylogenetic community structures are known to carry information on ecological assemblages because the relatedness of members in a community suggests similar ecological requirements and functions of phylogenetically related taxa [87,88]. For example, Kohler et al. [89] and Nagy et al. [90] showed phylogenetic relatedness of fungal orders based on gene counts for certain saprotrophic traits such as degradation of cellulose or lignin. Using gene counts, a large fraction of variance of potential fungal traits for N and P transformation was explained at the level of the subphylum or phylum [91]. Mycorrhizal species (identified on roots by morphotyping-Sanger sequencing) showed stronger phylogenetic clustering in drier and acidic habitats than under cooler and humid soil conditions [37]. Here, we discovered that distinct RAF orders showed a territorial behaviour as they were always predominant in a certain soil stratum, irrespective of differences in the environmental conditions among the region, whereas other fungal orders showed flexible behaviour with changing effect sizes depending on the regional habitat conditions.

Our results indicate that the behaviour whether a fungal group was territorial or flexible distinguished saprotrophic and symbiotrophic orders in RAF assemblages. Shared territorial mycorrhizal fungal orders occurred in the organic layer (Boletales) and in mineral soil (Russulales and Cantharellales), whereas we did not find any saprotrophic fungal order nor any saprotrophic indicator species that was shared in the mineral soil among the three regions. As expected the saprotrophic groups were either flexible or enriched in the organic layer, the latter group including Polyporales, Pleosporales, and Sordariales. Polyporales (some causing brown-rot of timber) are well known for their efficient lignolytic capabilities to degrade deadwood [92]. They were also enriched in the litter and organic soil surface in other temperate forests [21,69,93]. At the species level, *Calycellina fagina* was a shared indicator taxon in the organic layer across all study regions. Members of this genus are usually growing on dead-wood and plant matter in forest floors and play a role in decomposition processes [94,95].

Thelephorales, Sebacinales (both ectomycorrhizal fungi), Agaricales and Helotiales (mainly mixed symbiotrophic/saprotrophic fungi with small contributions (<4%) of pathogenic fungi) were major fungal orders in RAF assemblages with flexible effect sizes. This result indicates that dominant members of these orders are driven by the specific habitat conditions in each region. On the contrary, Russulales were consistently enriched in the mineral soil, suggesting territorial behaviour of the members of this order. Russulales are highly abundant ectomycorrhizal species in temperate beech forests [37,84,96]. All known members of the Russulales exhibit a hydrophilic contact exploration type [97], absorbing nutrients from the surrounding soil [98]. At the taxon level, *Russula* sp. was a shared indicator species on roots in mineral soil across all regions. Whether the availabilities of mineral nutrients foster root colonization with Russulales is unclear because fertilization experiments with P or N showed divergent effects on the abundance of Russulales (P fertilization: decrease [21,99]), and no effect of N fertilization in boreal forests [100,101]). Russulales have been classified as nitrophilic fungi but they are also capable of producing extracellular enzymes that could degrade organic matter in litter and soil [97]. Recent genome analyses indicated that they lost most enzymes required for cell wall degradation but maintained Mn peroxidases and chitinases [102]. Their involvement in decomposition is still questionable since they are relatively inactive in the period of organic matter decomposition [103]. Similar to Russulales, we found positive effect sizes for Cantharellales in the mineral layer. Members of this order also contain class II peroxidases capable of degrading complex organic compounds [89,104]. Perhaps these enzymes are beneficial for degradation of recalcitrant organic compounds still present in the mineral soil.

Boletales were the only mycorrhizal fungal order that showed significantly higher abundance in the RAF of the organic layer than in mineral soil across all regions. The relative abundance of Boletales increased after P fertilization [21]. In our studied forest soils, P_sol_ and N were higher in the organic layer than in mineral soil. Many Boletales species are characterised by long-distance rhizomorphs and can explore soil far beyond the rhizosphere [97]. Therefore, Boletales are considered as beneficial in nutrient-limited forest ecosystems. They can access distant resources [105] and, thus, meet the P demand of trees [106]. Almeida et al. [106] detected an increase in hyphal biomass of *Imleria badia* (Boletales) that accessed apatite (a recalcitrant P source) in N-fertilized soil but not in N-fertilized soil amended with soluble P sources, underpinning a role of these taxa for P nutrition.

At the species level, we detected *Laccaria amethystina* (Agaricales) as an indicator taxon on roots in the organic layer across all three regions, emphasizing the wide spread of this ectomycorrhizal fungal species across temperate forest ecosystems. *Laccaria amethystina* is among the most common species that colonize roots of oak and beech tree species in temperate deciduous and deciduous-coniferous forest ecosystem in Europe [107]. Besides *Laccaria amethystina*, related species such as *Laccaria maritima* were detected on beech roots in our studied forests [37,84]. Species from the Laccaria genus (e.g., *Laccaria bicolor*) have little capacity to degrade organic matter [108], which renders the occurrence of *Laccaria amethystina* as an indicator species in the RAF community in organic soil surprising. However, *Laccaria amethystina* is known as an ammonia utilizing fungus [109]. Therefore, we speculate that *Laccaria* sp. in the organic layer may benefit from N released by the degradation of organic material.

The consistent enrichment patterns of distinct RAF orders and species in response to soil layers across the regions support that ecological niche partitioning strongly influenced the differentiation of root-associated fungal community structures. Our results imply that RAF assembly entails two strategies encompassing flexible and territorial habitat colonization by different fungal taxa. However, it is important to take into account that this aggregated behaviour does not reflect collective response patterns of the species within a given order. This caveat is reflected by the identification of 39 saprotrophic and 63 symbiotrophic indicator taxa among which only three were confined to a distinct soil stratum and were shared among the regions. Other indicator taxa occurred only in one distinct region or were shared among the regions with higher similarities in soil resources [36]. Taking together individual variation of taxa in the RAF assemblage and relatively stable regional responses at a higher phylogenetic level, we suggest that this behaviour could lead to emergent properties of ecosystems that are more than the sum of their individuals.

## 5. Conclusions

Our results support the ecological concept that resource partitioning and phylogenetically conserved properties determine the ecological communities. A distinct response of RAF orders and indicator taxa that were specific to soil layer and region indicated that habitat conditions strongly influence the differentiation of the RAF community structure. The results also support a phylogenetic signature for niche partitioning since several fungal orders were enriched in distinct soil layers across a large-scale biogeographic gradient, irrespective of the habitat conditions. Saprotrophic fungal orders and indicator taxa were preferentially enriched in the organic layer and mycorrhizal orders and species in the mineral soil. However, our knowledge is still limited on fungal traits and fungal nutritional preferences. To better understand the role of RAF in shaping ecosystem properties, further studies on the interaction of mycorrhizal and saprotrophic fungi are required.

## Figures and Tables

**Table 1:** Key site characteristics of the three biogeographic regions in the Biodiversity Exploratories. Schwäbische Alb = ALB, Hainich-Dün = HAI and Schorfheide-Chorin = SCH. Data were compiled from [20,34,35,36,38].

Parameters	ALB	HAI	SCH
Location	Southwest Germany	Central Germany	Northeast Germany
Size (km^2^)	422	1300	1300
Geology	Calcareous bedrock with karst phenomena	Calcareous bedrock	Young glacial landscape
Altitude a.s.l (m)	460–860	285–550	3–140
Longitudes east to west (decimal degree)	9.58024–9.02362	10.77917–10.17332	14.14796–13.39094
Latitude north to south (decimal degree)	48.53435–48.34996	51.37872–50.93735	53.22390–52.79023
Mean annual temperature (°C)	6.0–7.0	6.5–8.0	8.0–8.5
Mean sum of annual precipitation (mm)	700–1000	500–800	500–600
Plot size (m)	100 × 100	100 × 100	100 × 100
Soil types	Cambisol (eutric) and Leptosol	Luvisol	Cambisol (dystric)
Mean soil pH	5.23 ± 0.10	4.80 ± 0.12	3.55 ± 0.02
Mean sand content (g kg^−1^)	59.60 ± 9.84	58.00 ± 9.84	871 ± 9.84
Mean silt content (g kg^−1^)	444 ± 15.70	642 ± 15.70	84.80 ± 15.70
Mean clay content (g kg^−1^)	496 ± 14.40	301 ± 14.00	44.8 ± 14.40
Main tree species	Beech (*Fagus sylvatica*) Spruce (*Picea abies)*	Beech (*Fagus sylvatica*) Spruce (*Picea abies*)	Beech (*Fagus sylvatica*) Pine (*Pinus sylvstris*) Oak (*Quercus* sp.)

**Table 2:** Soil chemical properties in different soil layers and regions. ALB = Schwäbische Alb, HAI = Hainich-Dün, SCH = Schorfheide-Chorin. Carbon (C) (g kg^−1^ DW); nitrogen (N) (g kg^−1^ DW); ratio of CN; phosphorus (P_sol_) (mg kg^−1^ DW); Cations: sum of potassium (K), calcium (Ca) and magnesium (Mg) (mmol kg^−1^ DW). Data indicate means ± SE (*n* = 50). Linear models were used to compare the means of the element between the regions. Significant differences of the means are shown in bold. Pairwise differences of the nutrient elements between organic layer and mineral soil were compared using a *post hoc test* (HSD Tukey’s honestly significant difference). Different letters denote significant differences between soil layers and within the regions.

	ALB		HAI		SCH		Organic		Mineral	
	Organic	Mineral	Organic	Mineral	Organic	Mineral	*F*	*p*	*F*	*p*
C	315.47 ± 6.65 (e)	58.53 ± 1.88 (c)	358.57 ± 7.80 (f)	40.86 ± 1.72 (b)	268.81 ± 8.64 (d)	22.93 ± 0.71 (a)	33.62	**<0.001**	135.97	**<0.001**
N	14.20 ± 0.34 (e)	4.33 ± 0.13 (c)	15.94 ± 0.37 (e)	2.98 ± 0.12 (b)	11.80 ± 0.27 (d)	1.21 ± 0.05 (a)	39.93	**<0.001**	211.22	**<0.001**
C:N	22.39 ± 0.34 (c)	13.54 ± 0.14 (a)	22.62 ± 0.30 (c)	13.74 ± 0.16 (a)	22.71 ± 0.43 (c)	19.48 ± 0.46 (b)	0.21	0.810	133.57	**<0.001**
P_sol_	230.22 ± 11.44 (d)	87.00 ± 8.06 (ab)	248.01 ± 11.88 (d)	58.03 ± 8.40 (a)	168.49 ± 9.67 (c)	119.87 ± 6.22 (b)	14.58	**<0.001**	16.31	**<0.001**
Cations	506.90 ± 24.95 (d)	370.14 ± 18.80 (c)	485.26 ± 14.76 (d)	334.77 ± 27.18 (c)	138.68 ± 7.01 (b)	48.38 ± 2.06 (a)	144.01	**<0.001**	85.18	**<0.001**

## Data Availability

All data are available in the BExIS database (https://www.bexis.uni-jena.de) under the following accession numbers (data owner): Soil chemistry—26228 and 26229 (Polle), root-associated fungal taxonomic and functional assignment—30973 and 30974 (Polle) and total fine root dry mass in organic layer and mineral topsoil—31048 and 31049 (Polle).

## Supplementary Materials

The following are available online at https://www.mdpi.com/article/10.3390/microorganisms9102131/s1, Figure S1: Relative abundance of root-associated fungi compiled at the order level in the organic layer and mineral soil and in different regions. Table S1: Location of the 150 study plots and fine root biomass. Table S2: Observed species richness (OTU-based), estimated richness (Chao1), Shannon diversity (H′) and evenness (*E_H_*) of root-associated fungi in different soil layers across three biogeographic regions. Table S3: Beta–diversity of root-associated fungal assemblages of all (OTUs), symbiotrophic (SYM), and saprotrophic (SAP) fungi between organic layer and mineral soil. Table S4: Relative abundance, number of sequences and number of taxa (OTU-based) of the root-associated fungal orders present in the organic layer and mineral soil in the Biodiversity Exploratory forest plots. Table S5: Classification of the top nineteen root-associated fungal orders according to functional groups. Table S6: Root-associated symbiotrophic and saprotrophic indicator taxa in the organic layer and mineral soil in three biogeographic regions.

## References

[1] Heijden M.G.A.V.D., Bardgett R.D., Straalen N.M.V. (2008). The Unseen Majority: Soil Microbes as Drivers of Plant Diversity and Productivity in Terrestrial Ecosystems. Ecol. Lett..

[2] Blackwell M. (2011). The Fungi: 1, 2, 3 … 5.1 Million Species?. Am. J. Bot..

[3] Baldrian P. (2017). Microbial Activity and the Dynamics of Ecosystem Processes in Forest Soils. Curr. Opin. Microbiol..

[4] García-Guzmán G., Heil M. (2014). Life Histories of Hosts and Pathogens Predict Patterns in Tropical Fungal Plant Diseases. New Phytol..

[5] Orwin K.H., Kirschbaum M.U.F., John M.G.S., Dickie I.A. (2011). Organic Nutrient Uptake by Mycorrhizal Fungi Enhances Ecosystem Carbon Storage: A Model-Based Assessment. Ecol. Lett..

[6] Hubbel S.P. (2001). The Unified Neutral Theory of Biodiversity and Biogeography (MPB-32).

[7] Cline L.C., Zak D.R. (2014). Dispersal Limitation Structures Fungal Community Assembly in a Long-Term Glacial Chronosequence. Environ. Microbiol..

[8] Tedersoo L., Bahram M., Põlme S., Kõljalg U., Yorou N.S., Wijesundera R., Ruiz L.V., Vasco-Palacios A.M., Thu P.Q., Suija A. (2014). Global Diversity and Geography of Soil Fungi. Science.

[9] Kraft N.J.B., Adler P.B., Godoy O., James E.C., Fuller S., Levine J.M. (2015). Community Assembly, Coexistence and the Environmental Filtering Metaphor. Funct. Ecol..

[10] Bahram M., Hildebrand F., Forslund S.K., Anderson J.L., Soudzilovskaia N.A., Bodegom P.M., Bengtsson-Palme J., Anslan S., Coelho L.P., Harend H. (2018). Structure and Function of the Global Topsoil Microbiome. Nature.

[11] Birkhofer K., Schöning I., Alt F., Herold N., Klarner B., Maraun M., Marhan S., Oelmann Y., Wubet T., Yurkov A. (2012). General Relationships between Abiotic Soil Properties and Soil Biota across Spatial Scales and Different Land-Use Types. PLoS ONE.

[12] Wubet T., Christ S., Schöning I., Boch S., Gawlich M., Schnabel B., Fischer M., Buscot F. (2012). Differences in Soil Fungal Communities between European Beech (Fagus Sylvatica L.) Dominated Forests Are Related to Soil and Understory Vegetation. PLoS ONE.

[13] Wang M., Shi S., Lin F., Jiang P. (2014). Response of the Soil Fungal Community to Multi-Factor Environmental Changes in a Temperate Forest. Appl. Soil Ecol..

[14] Goldmann K., Schöning I., Buscot F., Wubet T. (2015). Forest Management Type Influences Diversity and Community Composition of Soil Fungi across Temperate Forest Ecosystems. Front. Microbiol..

[15] Vandenkoornhuyse P., Quaiser A., Duhamel M., Van A.L., Dufresne A. (2015). The Importance of the Microbiome of the Plant Holobiont. New Phytol..

[16] Vandenkoornhuyse P., Baldauf S.L., Leyval C., Straczek J., Young J.P.W. (2002). Extensive Fungal Diversity in Plant Roots. Science.

[17] Goldmann K., Schröter K., Pena R., Schöning I., Schrumpf M., Buscot F., Polle A., Wubet T. (2016). Divergent Habitat Filtering of Root and Soil Fungal Communities in Temperate Beech Forests. Sci. Rep..

[18] Schröter K., Wemheuer B., Pena R., Schöning I., Ehbrecht M., Schall P., Ammer C., Daniel R., Polle A. (2019). Assembly Processes of Trophic Guilds in the Root Mycobiome of Temperate Forests. Mol. Ecol..

[19] Carteron A., Beigas M., Joly S., Turner B.L., Laliberté E. (2021). Temperate Forests Dominated by Arbuscular or Ectomycorrhizal Fungi Are Characterized by Strong Shifts from Saprotrophic to Mycorrhizal Fungi with Increasing Soil Depth. Microb. Ecol..

[20] Nguyen D.Q., Schneider D., Brinkmann N., Song B., Janz D., Schöning I., Daniel R., Pena R., Polle A. (2020). Soil and Root Nutrient Chemistry Structure Root-Associated Fungal Assemblages in Temperate Forests. Environ. Microbiol..

[21] Clausing S., Likulunga L.E., Janz D., Feng H.Y., Schneider D., Daniel R., Krüger J., Lang F., Polle A. (2021). Impact of Nitrogen and Phosphorus Addition on Resident Soil and Root Mycobiomes in Beech Forests. Biol. Fertil. Soils.

[22] Lindahl B.D., Ihrmark K., Boberg J., Trumbore S.E., Högberg P., Stenlid J., Finlay R.D. (2007). Spatial Separation of Litter Decomposition and Mycorrhizal Nitrogen Uptake in a Boreal Forest. New Phytol..

[23] Clemmensen K.E., Finlay R.D., Dahlberg A., Stenlid J., Wardle D.A., Lindahl B.D. (2015). Carbon Sequestration Is Related to Mycorrhizal Fungal Community Shifts during Long-Term Succession in Boreal Forests. New Phytol..

[24] Toju H., Kishida O., Katayama N., Takagi K. (2016). Networks Depicting the Fine-Scale Co-Occurrences of Fungi in Soil Horizons. PLoS ONE.

[25] Schlatter D.C., Kahl K., Carlson B., Huggins D.R., Paulitz T. (2018). Fungal Community Composition and Diversity Vary with Soil Depth and Landscape Position in a No-till Wheat-Based Cropping System. FEMS Microbiol. Ecol..

[26] Asplund J., Kauserud H., Ohlson M., Nybakken L. (2019). Spruce and Beech as Local Determinants of Forest Fungal Community Structure in Litter, Humus and Mineral Soil. FEMS Microbiol. Ecol..

[27] Jobbágy E.G., Jackson R.B. (2001). The Distribution of Soil Nutrients with Depth: Global Patterns and the Imprint of Plants. Biogeochemistry.

[28] Herold N., Schöning I., Berner D., Haslwimmer H., Kandeler E., Michalzik B., Schrumpf M. (2014). Vertical Gradients of Potential Enzyme Activities in Soil Profiles of European Beech, Norway Spruce and Scots Pine Dominated Forest Sites. Pedobiologia.

[29] Rosling A., Landeweert R., Lindahl B.D., Larsson K.-H., Kuyper T.W., Taylor A.F.S., Finlay R.D. (2003). Vertical Distribution of Ectomycorrhizal Fungal Taxa in a Podzol Soil Profile. New Phytol..

[30] Clemmensen K.E., Bahr A., Ovaskainen O., Dahlberg A., Ekblad A., Wallander H., Stenlid J., Finlay R.D., Wardle D.A., Lindahl B.D. (2013). Roots and Associated Fungi Drive Long-Term Carbon Sequestration in Boreal Forest. Science.

[31] Taylor D.L., Bruns T.D. (1999). Community Structure of Ectomycorrhizal Fungi in a Pinus Muricata Forest: Minimal Overlap between the Mature Forest and Resistant Propagule Communities. Mol. Ecol..

[32] Dickie I.A., Xu B., Koide R.T. (2002). Vertical Niche Differentiation of Ectomycorrhizal Hyphae in Soil as Shown by T-RFLP Analysis. New Phytol..

[33] Genney D.R., Anderson I.C., Alexander I.J. (2006). Fine-Scale Distribution of Pine Ectomycorrhizas and Their Extramatrical Mycelium. New Phytol..

[34] Fischer M., Bossdorf O., Gockel S., Hänsel F., Hemp A., Hessenmöller D., Korte G., Nieschulze J., Pfeiffer S., Prati D. (2010). Implementing Large-Scale and Long-Term Functional Biodiversity Research: The Biodiversity Exploratories. Basic Appl. Ecol..

[35] Solly E.F., Schöning I., Boch S., Kandeler E., Marhan S., Michalzik B., Müller J., Zscheischler J., Trumbore S.E., Schrumpf M. (2014). Factors Controlling Decomposition Rates of Fine Root Litter in Temperate Forests and Grasslands. Plant Soil.

[36] Gan H.Y., Schöning I., Schall P., Ammer C., Schrumpf M. (2020). Soil Organic Matter Mineralization as Driven by Nutrient Stoichiometry in Soils Under Differently Managed Forest Stands. Front. For. Glob. Chang..

[37] Pena R., Lang C., Lohaus G., Boch S., Schall P., Schöning I., Ammer C., Fischer M., Polle A. (2017). Phylogenetic and Functional Traits of Ectomycorrhizal Assemblages in Top Soil from Different Biogeographic Regions and Forest Types. Mycorrhiza.

[38] Leuschner C., Ellenberg H. (2017). Ecology of Central European Forests: Vegetation Ecology of Central Europe.

[39] Bray R.H., Kurtz L.T. (1945). Determination of Total, Organic, and Available Forms of Phosphorus in Soils. Soil Sci..

[40] Heinrichs H., Brumsack H.-J., Loftfield N., König N. (1986). Verbessertes Druckaufschlußsystem Für Biologische Und Anorganische Materialien. Z. Für Pflanz. Und Bodenkd..

[41] Horton T.R., Bruns T.D. (2001). The Molecular Revolution in Ectomycorrhizal Ecology: Peeking into the Black-Box. Mol. Ecol..

[42] Toju H., Tanabe A.S., Yamamoto S., Sato H. (2012). High-Coverage ITS Primers for the DNA-Based Identification of Ascomycetes and Basidiomycetes in Environmental Samples. PLoS ONE.

[43] White T.J., Bruns T., Lee S., Taylor J. (1990). Amplification and Direct Sequencing of Fungal Ribosomal RNA Genes for Phylogenetics. PCR Protocols.

[44] Von Hoyningen-Huene A.J.E., Schneider D., Fussmann D., Reimer A., Arp G., Daniel R. (2019). Bacterial Succession along a Sediment Porewater Gradient at Lake Neusiedl in Austria. Sci. Data.

[45] Zhang J., Kobert K., Flouri T., Stamatakis A. (2014). PEAR: A Fast and Accurate Illumina Paired-End ReAd MergeR. Bioinformatics.

[46] Martin M. (2011). Cutadapt Removes Adapter Sequences from High-Throughput Sequencing Reads. EMBnet. J..

[47] Callahan B.J., McMurdie P.J., Holmes S.P. (2017). Exact Sequence Variants Should Replace Operational Taxonomic Units in Marker-Gene Data Analysis. ISME J..

[48] Edgar R.C. (2010). Search and Clustering Orders of Magnitude Faster than BLAST. Bioinformatics.

[49] Edgar R.C. (2016). UNOISE2: Improved Error-Correction for Illumina 16S and ITS Amplicon Sequencing. bioRxiv.

[50] Kõljalg U., Nilsson R.H., Abarenkov K., Tedersoo L., Taylor A.F.S., Bahram M., Bates S.T., Bruns T.D., Bengtsson-Palme J., Callaghan T.M. (2013). Towards a Unified Paradigm for Sequence-Based Identification of Fungi. Mol. Ecol..

[51] McDonald D., Clemente J.C., Kuczynski J., Rideout J.R., Stombaugh J., Wendel D., Wilke A., Huse S., Hufnagle J., Meyer F. (2012). The Biological Observation Matrix (BIOM) Format or: How I Learned to Stop Worrying and Love the Ome-Ome. GigaScience.

[52] Altschul S.F., Gish W., Miller W., Myers E.W., Lipman D.J. (1990). Basic Local Alignment Search Tool. J. Mol. Biol..

[53] Andersen K.S., Kirkegaard R.H., Karst S.M., Albertsen M. (2018). Ampvis2: An R Package to Analyse and Visualise 16S RRNA Amplicon Data. bioRxiv.

[54] Nguyen N.H., Song Z., Bates S.T., Branco S., Tedersoo L., Menke J., Schilling J.S., Kennedy P.G. (2016). FUNGuild: An Open Annotation Tool for Parsing Fungal Community Datasets by Ecological Guild. Fungal Ecol..

[55] R Development Core Team (2020). R: The R Project for Statistical Computing: R Foundation for Statistical Computing.

[56] Bates D., Mächler M., Bolker B., Walker S. (2015). Fitting Linear Mixed-Effects Models Using Lme4. J. Stat. Softw..

[57] Hothorn T., Bretz F., Westfall P. (2008). Simultaneous Inference in General Parametric Models. Biom. J..

[58] Oksanen J., Blanchet F.G., Friendly M., Kindt R., Legendre P., McGlinn D., Minchin P.R., O’Hara R.B., Simpson G.L., Solymos P. (2013). Vegan: Community Ecology Package. https://cran.ism.ac.jp/web/packages/vegan/vegan.pdf.

[59] Ho N.T., Li F., Wang S., Kuhn L. (2019). MetamicrobiomeR: An R Package for Analysis of Microbiome Relative Abundance Data Using Zero-Inflated Beta GAMLSS and Meta-Analysis across Studies Using Random Effects Models. BMC Bioinform..

[60] Baselga A., Orme C.D.L. (2012). Betapart: An R Package for the Study of Beta Diversity. Methods Ecol. Evol..

[61] Dormann C.F., Fründ J., Blüthgen N., Gruber B. (2009). Indices, Graphs and Null Models: Analyzing Bipartite Ecological Networks. Open Ecol. J..

[62] Cáceres M.D., Legendre P. (2009). Associations between Species and Groups of Sites: Indices and Statistical Inference. Ecology.

[63] Wickham H. (2009). Ggplot2: Elegant Graphics for Data Analysis.

[64] Shigyo N., Umeki K., Hirao T. (2019). Seasonal Dynamics of Soil Fungal and Bacterial Communities in Cool-Temperate Montane Forests. Front. Microbiol..

[65] Voříšková J., Brabcová V., Cajthaml T., Baldrian P. (2014). Seasonal Dynamics of Fungal Communities in a Temperate Oak Forest Soil. New Phytol..

[66] Brabcová V., Nováková M., Davidová A., Baldrian P. (2016). Dead Fungal Mycelium in Forest Soil Represents a Decomposition Hotspot and a Habitat for a Specific Microbial Community. New Phytol..

[67] Bödeker I.T.M., Lindahl B.D., Olson Å., Clemmensen K.E. (2016). Mycorrhizal and Saprotrophic Fungal Guilds Compete for the Same Organic Substrates but Affect Decomposition Differently. Funct. Ecol..

[68] Santalahti M., Sun H., Jumpponen A., Pennanen T., Heinonsalo J. (2016). Vertical and Seasonal Dynamics of Fungal Communities in Boreal Scots Pine Forest Soil. FEMS Microbiol. Ecol..

[69] Peršoh D., Stolle N., Brachmann A., Begerow D., Rambold G. (2018). Fungal Guilds Are Evenly Distributed along a Vertical Spruce Forest Soil Profile While Individual Fungi Show Pronounced Niche Partitioning. Mycol. Prog..

[70] Tedersoo L., Kõljalg U., Hallenberg N., Larsson K.-H. (2003). Fine Scale Distribution of Ectomycorrhizal Fungi and Roots across Substrate Layers Including Coarse Woody Debris in a Mixed Forest. New Phytol..

[71] Mrak T., Hukić E., Štraus I., Unuk Nahberger T., Kraigher H. (2020). Ectomycorrhizal Community Composition of Organic and Mineral Soil Horizons in Silver Fir (Abies Alba Mill.) Stands. Mycorrhiza.

[72] Grinhut T., Hadar Y., Chen Y. (2007). Degradation and Transformation of Humic Substances by Saprotrophic Fungi: Processes and Mechanisms. Fungal Biol. Rev..

[73] Baldrian P., Boddy L., Frankland J.C., van West P. (2008). Chapter 2 Enzymes of saprotrophic basidiomycetes. Ecology of Saprotrophic Basidiomycetes.

[74] Colpaert J.V., Laere A.V. (1996). A Comparison of the Extracellular Enzyme Activities of Two Ectomycorrhizal and a Leaf-Saprotrophic Basidiomycete Colonizing Beech Leaf Litter. New Phytol..

[75] Santalahti M., Putkinen A., Adamczyk S., Sun H., Heinonsalo J. (2019). Restriction of plant roots in boreal forest organic soils affects the microbial community but does not change the dominance from ectomycorrhizal to saprotrophic fungi. FEMS Microbiol. Ecol..

[76] Chase J.M. (2007). Drought Mediates the Importance of Stochastic Community Assembly. Proc. Natl. Acad. Sci. USA.

[77] Marx D.H., Bryan W.C., Davey C.B. (1970). Influence of Temperature on Aseptic Synthesis of Ectomycorrhizae by Thelephora Terrestris and Pisolithus Tinctorius on Loblolly Pine. For. Sci..

[78] Lazarevic J., Stojicic D., Keca N. (2016). Effects of Temperature, PH and Carbon and Nitrogen Sources on Growth of in Vitro Cultures of Ectomycorrhizal Isolates from Pinus Heldreichii Forest. For. Syst..

[79] Leberecht M., Tu J., Polle A. (2016). Acid and Calcareous Soils Affect Nitrogen Nutrition and Organic Nitrogen Uptake by Beech Seedlings (Fagus Sylvatica L.) under Drought, and Their Ectomycorrhizal Community Structure. Plant Soil.

[80] Mucha J., Peay K.G., Smith D.P., Reich P.B., Stefański A., Hobbie S.E. (2018). Effect of Simulated Climate Warming on the Ectomycorrhizal Fungal Community of Boreal and Temperate Host Species Growing Near Their Shared Ecotonal Range Limits. Microb. Ecol..

[81] Taniguchi T., Kitajima K., Douhan G.W., Yamanaka N., Allen M.F. (2018). A Pulse of Summer Precipitation after the Dry Season Triggers Changes in Ectomycorrhizal Formation, Diversity, and Community Composition in a Mediterranean Forest in California, USA. Mycorrhiza.

[82] Kranabetter J.M., Durall D.M., MacKenzie W.H. (2009). Diversity and Species Distribution of Ectomycorrhizal Fungi along Productivity Gradients of a Southern Boreal Forest. Mycorrhiza.

[83] Ishida T.A., Nara K., Hogetsu T. (2007). Host Effects on Ectomycorrhizal Fungal Communities: Insight from Eight Host Species in Mixed Conifer–Broadleaf Forests. New Phytol..

[84] Lang C., Seven J., Polle A. (2011). Host Preferences and Differential Contributions of Deciduous Tree Species Shape Mycorrhizal Species Richness in a Mixed Central European Forest. Mycorrhiza.

[85] Van der Linde S., Suz L.M., Orme C.D.L., Cox F., Andreae H., Asi E., Atkinson B., Benham S., Carroll C., Cools N. (2018). Environment and Host as Large-Scale Controls of Ectomycorrhizal Fungi. Nature.

[86] Ballauff J., Schneider D., Edy N., Irawan B., Daniel R., Polle A. (2021). Shifts in Root and Soil Chemistry Drive the Assembly of Belowground Fungal Communities in Tropical Land-Use Systems. Soil Biol. Biochem..

[87] Cavender-Bares J., Kozak K.H., Fine P.V.A., Kembel S.W. (2009). The Merging of Community Ecology and Phylogenetic Biology. Ecol. Lett..

[88] Pausas J.G., Verdú M. (2010). The Jungle of Methods for Evaluating Phenotypic and Phylogenetic Structure of Communities. BioScience.

[89] Kohler A., Kuo A., Nagy L.G., Morin E., Barry K.W., Buscot F., Canbäck B., Choi C., Cichocki N., Clum A. (2015). Convergent Losses of Decay Mechanisms and Rapid Turnover of Symbiosis Genes in Mycorrhizal Mutualists. Nat. Genet..

[90] Nagy L.G., Riley R., Tritt A., Adam C., Daum C., Floudas D., Sun H., Yadav J.S., Pangilinan J., Larsson K.-H. (2016). Comparative Genomics of Early-Diverging Mushroom-Forming Fungi Provides Insights into the Origins of Lignocellulose Decay Capabilities. Mol. Biol. Evol..

[91] Treseder K.K., Lennon J.T. (2015). Fungal Traits That Drive Ecosystem Dynamics on Land. Microbiol. Mol. Biol. Rev..

[92] Binder M., Justo A., Riley R., Salamov A., Lopez-Giraldez F., Sjökvist E., Copeland A., Foster B., Sun H., Larsson E. (2013). Phylogenetic and Phylogenomic Overview of the Polyporales. Mycologia.

[93] Žifčáková L., Větrovský T., Howe A., Baldrian P. (2016). Microbial Activity in Forest Soil Reflects the Changes in Ecosystem Properties between Summer and Winter. Environ. Microbiol..

[94] Cannon P.F., Kirk P.M. (2007). Fungal Families of the World.

[95] Badali H., Gueidan C., Najafzadeh M.J., Bonifaz A., van den Ende A.H.G.G., de Hoog G.S. (2008). Biodiversity of the Genus Cladophialophora. Stud. Mycol..

[96] Buée M., Vairelles D., Garbaye J. (2005). Year-Round Monitoring of Diversity and Potential Metabolic Activity of the Ectomycorrhizal Community in a Beech (Fagus Silvatica) Forest Subjected to Two Thinning Regimes. Mycorrhiza.

[97] Agerer R. (2001). Exploration Types of Ectomycorrhizae. Mycorrhiza.

[98] Pena R., Tejedor J., Zeller B., Dannenmann M., Polle A. (2013). Interspecific Temporal and Spatial Differences in the Acquisition of Litter-Derived Nitrogen by Ectomycorrhizal Fungal Assemblages. New Phytol..

[99] Mason L.M., Eagar A., Patel P., Blackwood C.B., DeForest J.L. (2021). Potential Microbial Bioindicators of Phosphorus Mining in a Temperate Deciduous Forest. J. Appl. Microbiol..

[100] Allison S.D., Hanson C.A., Treseder K.K. (2007). Nitrogen Fertilization Reduces Diversity and Alters Community Structure of Active Fungi in Boreal Ecosystems. Soil Biol. Biochem..

[101] Nicolás C., Almeida J.P., Ellström M., Bahr A., Bone S.E., Rosenstock N.P., Bargar J.R., Tunlid A., Persson P., Wallander H. (2017). Chemical Changes in Organic Matter after Fungal Colonization in a Nitrogen Fertilized and Unfertilized Norway Spruce Forest. Plant Soil.

[102] Looney B., Miyauchi S., Morin E., Drula E., Courty P.E., Kohler A., Kuo A., LaButti K., Pangilinan J., Lipzen A. (2021). Evolutionary Priming and Transition to the Ectomycorrhizal Habit in an Iconic Lineage of Mushroom-Forming Fungi: Is Preadaptation a Requirement?. bioRxiv.

[103] Baldrian P., Kolařík M., Štursová M., Kopecký J., Valášková V., Větrovský T., Žifčáková L., Šnajdr J., Rídl J., Vlček Č. (2012). Active and Total Microbial Communities in Forest Soil Are Largely Different and Highly Stratified during Decomposition. ISME J..

[104] Sinsabaugh R.L. (2010). Phenol Oxidase, Peroxidase and Organic Matter Dynamics of Soil. Soil Biol. Biochem..

[105] Hobbie E.A., Agerer R. (2010). Nitrogen Isotopes in Ectomycorrhizal Sporocarps Correspond to Belowground Exploration Types. Plant Soil.

[106] Almeida J.P., Rosenstock N.P., Forsmark B., Bergh J., Wallander H. (2019). Ectomycorrhizal Community Composition and Function in a Spruce Forest Transitioning between Nitrogen and Phosphorus Limitation. Fungal. Ecol..

[107] Mueller G.M., History F.M. (1992). Systematics of Laccaria (Agaricales) in the Continental United States and Canada, with Discussions on Extralimital Taxa and Descriptions of Extant Types.

[108] Martin F., Aerts A., Ahrén D., Brun A., Danchin E.G.J., Duchaussoy F., Gibon J., Kohler A., Lindquist E., Pereda V. (2008). The Genome of Laccaria Bicolor Provides Insights into Mycorrhizal Symbiosis. Nature.

[109] Imamura A. (2001). Report on Laccaria Amethystina, Newly Confirmed as an Ammonia Fungus. Mycoscience.

