# Supplementary material for: Soil Layers Matter: Vertical Stratification of Root-Associated Fungal Assemblages in Temperate Forests Reveals Differences in Habitat Colonization
Source: Microorganisms. 2021 Oct 11;9(10):2131. doi: 10.3390/microorganisms9102131 (PMC8537680; doi:10.3390/microorganisms9102131)
Supplement: Supplementary file 1 [file microorganisms-09-02131-s001.zip › microorganisms-1356626-supplementary.pdf]

Supplementary File

Soil layers matter: vertical stratification of root-associated fungal assemblages in temperate forests reveals differences in habitat colonization

Anis Mahmud Khokon [1], Dominik Schneider [2], Rolf Daniel [2] and Andrea Polle [1,*]

[1] Forest Botany and Tree Physiology, University of Göttingen, 37077 Göttingen, Germany; anis.khokon@gwdg.de (A.M.K.)

[2] Genomic and Applied Microbiology and Göttingen Genomics Laboratory, Institute of Microbiology and Genetics, University of Göttingen, 37077 Göttingen, Germany; dschnei1@gwdg.de (D.S.); rdaniel@gwdg.de (R.D.)

* Correspondence: apolle@gwdg.de (A.P.)

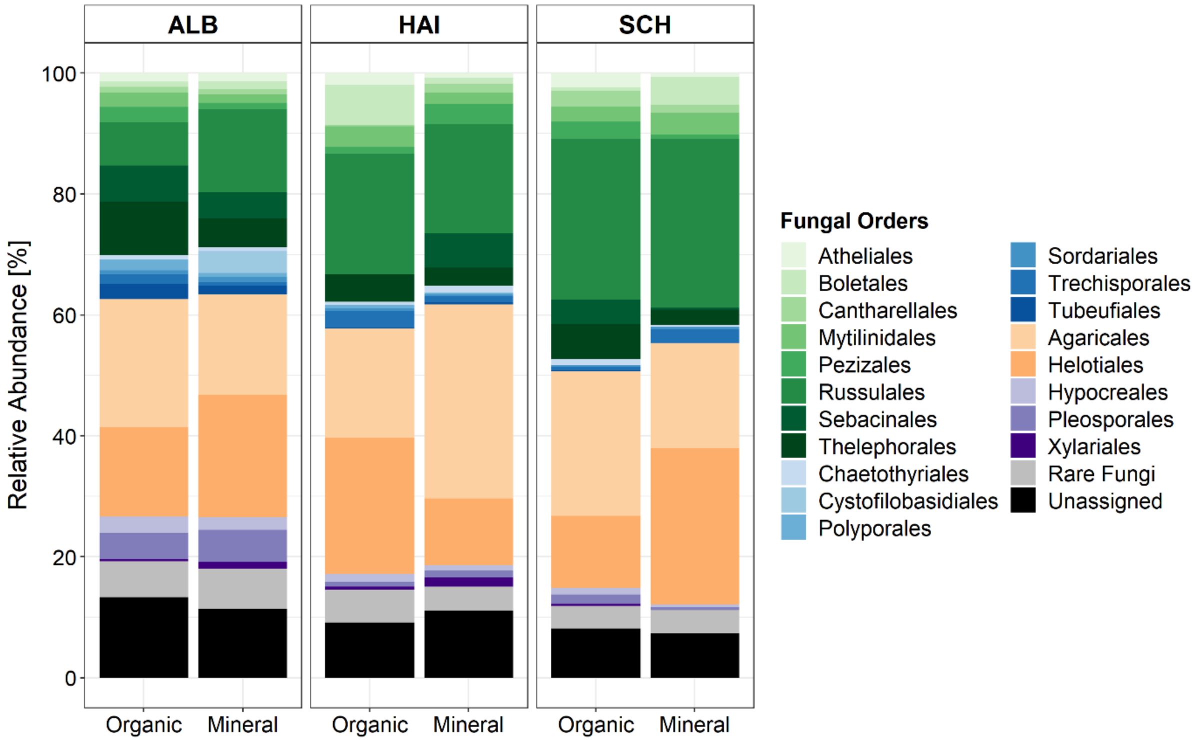

Figure S1: Relative abundance of root-associated fungi compiled at the order level in the organic layer and mineral soil and in different regions. ALB = Schwäbische Alb, HAI = Hainich-Dün and SCH = Schorfheide-Chorin. "Rare Fungi" (grey color): fungal orders with < 0.5% of the total number of sequences were summarized. Unassigned (black color): fungi that were not identified at the level of the fungal order were summarized. Fungal orders: green colours refer to symbiotrophic (SYM), blue to saprotrophic (SAP), orange to mixed SYM + SAP and purple to SAP + PAT fungal guilds.

Table S1: Location of the study plots in the Biodiversity Exploratories. Schwäbische Alb = ALB, Hainich-Dün = HAI and Schorfheide-Chorin = SCH, NA = not available. Total dry fine root biomass (g kg^{-1} soil) in organic layer and in mineral topsoil. Data were taken from BExIS datasets ID (1000, 31048 and 31049).

Plot ID	Regions	Latitude	Longitude	fine root biomass in organic layers	fine root biomass in mineral topsoil
ALB1	ALB	48.48	9.33	3.92	1.87
ALB2	ALB	48.38	9.35	2.88	1.56
ALB3	ALB	48.41	9.36	10.54	2.00
ALB4	ALB	48.4	9.24	1.22	2.89
ALB5	ALB	48.42	9.41	0.64	2.29
ALB6	ALB	48.39	9.45	2.84	2.09
ALB7	ALB	48.4	9.26	6.08	2.54
ALB8	ALB	48.38	9.38	3.08	2.72
ALB9	ALB	48.37	9.42	5.42	3.45
ALB10	ALB	48.38	9.39	4.05	1.58
ALB11	ALB	48.49	9.32	5.84	2.79
ALB12	ALB	48.37	9.25	6.59	1.62
ALB13	ALB	48.38	9.4	1.66	2.60
ALB14	ALB	48.38	9.34	1.26	1.65
ALB15	ALB	48.39	9.41	0.75	1.70
ALB16	ALB	48.39	9.44	9.48	3.65
ALB17	ALB	48.4	9.24	1.11	1.77
ALB18	ALB	48.37	9.23	1.25	2.35
ALB19	ALB	48.48	9.31	2.25	3.69
ALB20	ALB	48.37	9.32	0.30	2.01
ALB21	ALB	48.48	9.32	3.11	3.71
ALB22	ALB	48.38	9.45	0.98	3.11
ALB23	ALB	48.39	9.49	2.64	3.30
ALB24	ALB	48.48	9.29	2.55	4.44
ALB25	ALB	48.48	9.42	0.14	3.17
ALB26	ALB	48.42	9.44	1.02	1.88
ALB27	ALB	48.4	9.47	0.88	1.53
ALB28	ALB	48.49	9.31	3.93	2.05
ALB29	ALB	48.38	9.36	2.74	2.39
ALB30	ALB	48.37	9.37	0.49	4.02
ALB31	ALB	48.38	9.26	1.76	1.57
ALB32	ALB	48.41	9.37	2.88	6.85
ALB33	ALB	48.39	9.49	7.64	4.43
ALB34	ALB	48.39	9.5	6.40	2.39
ALB35	ALB	48.45	9.42	0.78	2.82
ALB36	ALB	48.38	9.22	1.17	4.00
ALB37	ALB	48.39	9.44	0.67	4.21
ALB38	ALB	48.38	9.45	2.06	2.25
ALB39	ALB	48.38	9.24	1.11	3.49
ALB40	ALB	48.5	9.35	0.52	3.96
ALB41	ALB	48.36	9.4	0.50	2.47
ALB42	ALB	48.4	9.45	1.15	NA

ALB43	ALB	48.38	9.38	1.91	1.07
ALB44	ALB	48.39	9.22	0.84	3.99
ALB45	ALB	48.39	9.5	3.04	1.92
ALB46	ALB	48.39	9.36	1.05	1.68
ALB47	ALB	48.42	9.41	3.84	2.82
ALB48	ALB	48.39	9.47	0.67	1.90
ALB49	ALB	48.45	9.48	0.34	3.57
ALB50	ALB	48.39	9.25	1.29	4.06
HAI1	HAI	51.19	10.32	5.35	1.30
HAI3	HAI	51.27	10.31	6.49	1.40
HAI4	HAI	51.37	10.53	3.72	4.17
HAI5	HAI	51.26	10.24	3.27	1.42
HAI6	HAI	51.27	10.24	3.98	1.95
HAI7	HAI	51.13	10.39	4.37	1.80
HAI8	HAI	51.36	10.52	1.74	1.86
HAI9	HAI	51.13	10.38	2.76	0.76
HAI10	HAI	51.09	10.46	0.93	1.01
HAI11	HAI	51.1	10.4	1.43	1.90
HAI12	HAI	51.1	10.46	1.77	1.11
HAI13	HAI	51.24	10.31	1.71	1.15
HAI14	HAI	51.37	10.26	4.31	3.56
HAI15	HAI	51.21	10.39	2.99	2.63
HAI16	HAI	51.18	10.37	8.12	3.53
HAI17	HAI	51.28	10.23	2.51	2.18
HAI18	HAI	51.34	10.37	8.47	6.19
HAI19	HAI	51.2	10.34	2.41	1.04
HAI20	HAI	51.33	10.36	3.09	2.13
HAI21	HAI	51.19	10.32	3.50	1.67
HAI22	HAI	51.34	10.36	9.02	1.74
HAI23	HAI	51.28	10.21	3.28	3.17
HAI24	HAI	51.05	10.49	1.79	1.31
HAI25	HAI	51.28	10.22	2.86	2.67
HAI26	HAI	51.34	10.51	2.97	1.78
HAI27	HAI	51.36	10.52	2.85	1.45
HAI28	HAI	51.36	10.53	1.13	0.82
HAI29	HAI	51.13	10.38	2.44	1.05
HAI30	HAI	51.13	10.38	3.02	0.97
HAI31	HAI	51.14	10.38	3.28	1.40
HAI32	HAI	51.14	10.36	3.18	1.36
HAI33	HAI	51.34	10.51	1.89	1.44
HAI34	HAI	51.08	10.45	0.73	1.61
HAI35	HAI	51.1	10.41	2.05	1.96
HAI36	HAI	51.11	10.41	1.34	2.60
HAI37	HAI	51.08	10.46	0.49	1.29
HAI38	HAI	51.08	10.46	2.25	1.67
HAI39	HAI	51.09	10.47	0.42	1.05
HAI40	HAI	51.08	10.5	0.56	1.93

HAI41	HAI	51.11	10.45	1.32	1.75
HAI42	HAI	51.1	10.46	0.76	1.00
HAI43	HAI	51.33	10.36	1.32	4.27
HAI44	HAI	51.33	10.37	9.26	4.81
HAI45	HAI	51.36	10.26	10.47	1.87
HAI46	HAI	51.05	10.48	0.91	1.55
HAI47	HAI	51.18	10.38	6.40	1.74
HAI48	HAI	51.14	10.39	1.89	1.43
HAI49	HAI	51.14	10.38	4.58	1.15
HAI50	HAI	51.09	10.46	0.60	1.76
HAI51	HAI	51.21	10.37	2.56	4.81
SCH1	SCH	52.9	13.85	11.15	0.49
SCH2	SCH	52.95	13.78	14.35	0.82
SCH3	SCH	52.92	13.64	24.73	0.35
SCH4	SCH	52.92	13.85	9.53	0.83
SCH5	SCH	53.06	13.89	17.17	NA
SCH6	SCH	52.91	13.84	14.09	1.55
SCH7	SCH	53.11	13.69	4.46	1.13
SCH8	SCH	53.19	13.93	1.91	1.30
SCH9	SCH	53.04	13.81	36.54	1.14
SCH10	SCH	53.09	13.64	4.70	0.49
SCH11	SCH	53.09	13.63	6.46	0.49
SCH12	SCH	53.09	13.7	11.46	0.69
SCH13	SCH	53.02	13.86	8.81	0.74
SCH14	SCH	52.92	13.75	5.58	0.29
SCH15	SCH	52.91	13.74	5.99	0.23
SCH16	SCH	53.08	13.86	21.54	0.68
SCH17	SCH	52.94	13.83	27.13	0.46
SCH18	SCH	52.86	13.92	19.92	0.68
SCH19	SCH	53.09	13.64	7.72	0.41
SCH20	SCH	53.09	13.64	13.44	1.09
SCH21	SCH	52.92	13.74	18.12	0.62
SCH22	SCH	53.06	13.91	2.76	0.54
SCH23	SCH	52.95	13.78	6.41	0.76
SCH24	SCH	52.89	13.97	5.28	0.82
SCH25	SCH	52.92	13.76	4.37	0.90
SCH26	SCH	52.92	13.75	4.04	0.54
SCH27	SCH	52.89	14	2.35	0.87
SCH28	SCH	52.88	13.66	3.66	0.38
SCH29	SCH	53.02	13.86	9.36	0.85
SCH30	SCH	53.06	13.92	3.82	0.71
SCH31	SCH	52.88	13.9	15.92	1.25
SCH32	SCH	53.06	13.93	4.00	1.61
SCH33	SCH	52.92	13.89	18.51	1.12
SCH34	SCH	52.9	13.85	20.01	0.58
SCH35	SCH	52.91	13.85	13.93	0.88
SCH36	SCH	52.95	13.75	10.84	1.53

SCH37	SCH	52.94	13.78	11.23	1.56
SCH38	SCH	52.89	13.67	5.85	0.77
SCH39	SCH	52.92	13.87	9.66	0.91
SCH40	SCH	52.91	13.86	12.21	0.71
SCH41	SCH	52.91	13.91	12.41	1.41
SCH42	SCH	52.9	13.91	10.03	1.83
SCH43	SCH	52.9	13.93	14.16	1.44
SCH44	SCH	52.92	13.86	6.83	1.50
SCH45	SCH	53.05	13.84	4.87	0.98
SCH46	SCH	53.07	13.78	2.62	0.85
SCH47	SCH	53.07	13.77	5.81	1.33
SCH48	SCH	53.05	13.84	2.73	0.68
SCH49	SCH	52.89	13.89	13.76	1.21
SCH50	SCH	53.04	13.79	3.85	1.51

Table S2: Observed species richness (OTU-based), estimated richness (Chao1), Shannon diversity (H′) and evenness (E_H) of root-associated fungi in different soil layers across three biogeographic regions. Symbiotrophic = SYM, saprotrophic = SAP, pathotrophic = PAT, ALB = Schwäbische Alb, HAI = Hainich-Dün, SCH = Schorfheide-Chorin. Data indicate means ± SE (n = 50). Linear models were used to compare the means of Chao1, Shannon and evenness between the regions. Generalized linear models were used to compare means of the observed species richness between the regions. Significant differences of the means are shown in bold. Different letters denote significant differences between soil layers and within the regions.

	**ALB**		**HAI**		**SCH**		***p*-value (regions)**	
	**Organic**	**Mineral**	**Organic**	**Mineral**	**Organic**	**Mineral**	**Organic**	**Mineral**
**All Fungi**								
OTU Richness	188.36 ± 8.70 (e)	146.26 ± 5.24 (b)	164.22 ± 8.16 (d)	156.84 ± 5.92 (c)	158.24 ± 4.61 (cd)	106.08 ± 5.90 (a)	**< 0.001**	**< 0.001**
Chao1	285.77 ± 13.07 (c)	223.99 ± 7.94 (b)	258.97 ± 12.29 (bc)	237.56 ± 8.67 (b)	243.76 ± 7.40 (b)	160.48 ± 9.17 (a)	**0.030**	**< 0.001**
Shannon (H')	2.93 ± 0.11 (b)	2.83 ± 0.08 (ab)	2.79 ± 0.11 (ab)	2.95 ± 0.07 (b)	2.98 ± 0.07 (b)	2.56 ± 0.08 (a)	0.360	**0.002**
Evenness (E_H)	0.12 ± 0.01 (a)	0.13 ± 0.01 (a)	0.12 ± 0.01 (a)	0.13 ± 0.01 (a)	0.13 ± 0.01 (a)	0.14 ± 0.01 (a)	0.141	0.175
**SYM**								
OTU Richness	24.90 ± 1.70 (b)	44.22 ± 2.11 (c)	22.54 ± 1.54 (b)	46.30 ± 2.35 (c)	19.34 ± 0.87 (a)	24.92 ± 1.90 (b)	**< 0.001**	**< 0.001**
Chao1	39.30 ± 3.52 (b)	61.13 ± 3.05 (c)	34.25 ± 2.78 (ab)	63.95 ± 3.34 (c)	24.72 ± 1.24 (a)	32.04 ± 2.50 (ab)	**< 0.001**	**< 0.001**
Shannon (H')	1.64 ± 0.08 (ab)	2.17 ± 0.08 (c)	1.45 ± 0.10 (a)	1.53 ± 0.07 (bc)	1.92 ± 0.09 (a)	1.57 ± 0.08 (a)	0.280	**< 0.001**
Evenness (E_H)	0.26 ± 0.02 (b)	0.24 ± 0.02 (b)	0.25 ± 0.02 (b)	0.17 ± 0.01 (a)	0.27 ± 0.01 (b)	0.24 ± 0.01 (ab)	0.751	**< 0.001**
**SAP**								
OTU Richness	47.80 ± 2.36 (d)	29.96 ± 1.45 (b)	40.68 ± 2.12 (c)	32.06 ± 1.41 (b)	42.42 ± 1.55 (c)	24.12 ± 1.38 (a)	**< 0.001**	**< 0.001**
Chao1	70.40 ± 4.08 (b)	46.85 ± 2.67 (a)	65.21 ± 3.89 (b)	47.92 ± 2.30 (a)	64.96 ± 2.89 (b)	38.38 ± 2.70 (a)	0.495	**0.017**
Shannon (H')	2.30 ± 0.10 (a)	1.95 ± 0.08 (a)	2.15 ± 0.12 (a)	2.20 ± 0.10 (a)	2.28 ± 0.10 (a)	1.95 ± 0.08 (a)	0.596	**0.070**
Evenness (E_H)	0.26 ± 0.02 (a)	0.28 ± 0.02 (a)	0.31 ± 0.03 (a)	0.36 ± 0.03 (a)	0.29 ± 0.02 (a)	0.36 ± 0.03 (a)	0.521	**0.019**
**PAT**								
OTU Richness	10.20 ± 0.64 (d)	4.56 ± 0.32 (b)	7.92 ± 0.51 (c)	5.30 ± 0.32 (b)	8.24 ± 0.59 (c)	3.18 ± 0.27 (a)	**< 0.001**	**< 0.001**
Chao1	14.71 ± 1.01 (c)	6.50 ± 0.60 (a)	13.33 ± 1.09 (bc)	7.37 ± 0.65 (a)	11.30 ± 0.89 (b)	4.52 ± 0.52 (a)	**0.056**	**0.003**
Shannon (H')	1.56 ± 0.07 (d)	1.05 ± 0.07 (b)	1.45 ± 0.08 (cd)	1.21 ± 0.07 (bc)	1.61 ± 0.08 (d)	0.74 ± 0.08 (a)	0.334	**< 0.001**
Evenness (E_H)	0.58 ± 0.03 (a)	0.76 ± 0.04 (b)	0.67 ± 0.04 (ab)	0.72 ± 0.03 (b)	0.72 ± 0.03 (b)	0.80 ± 0.04 (b)	**0.006**	0.263

Table S3: Beta diversity of root-associated fungal assemblages of all (OTUs), symbiotrophic (SYM), and saprotrophic (SAP) fungi between organic layer and mineral soil. Linear models were used to determine the extent of the fungal turnover (β_{SIM}), nestedness (β_{SNE}) and total beta-diversity (β_{SOR}) among the regions. Data indicate (n = 50). Significant differences of the means are shown in bold.

Fungal groups	Diversity groups	Sum sq	DF	*F*-value	*p*-value (regions)
**All fungi**	Turnover	0.755	2	33.221	**< 0.001**
	Nestedness	0.117	2	13.373	**< 0.001**
	Beta-diversity	0.279	2	20.482	**< 0.001**
**SYM**	Turnover	0.764	2	13.989	**< 0.001**
	Nestedness	0.159	2	6.557	**0.002**
	Beta-diversity	1.608	2	41.20	**< 0.001**
**SAP**	Turnover	0.509	2	14.122	**< 0.001**
	Nestedness	0.175	2	9.977	**< 0.001**
	Beta-diversity	0.101	2	5.636	**0.004**

Table S4: Relative abundance, number of sequences and number of taxa (OTU-based) of the root-associated fungal orders present in the organic layer and mineral soil in the Biodiversity Exploratory forest plots.

Fungal order	Taxa	Sequences	Relative abundance
Agaricales	300	251005	21.57
Russulales	294	219690	18.88
Helotiales	300	206307	17.73
Thelephorales	283	57089	4.91
Sebacinales	224	39445	3.39
Mytilinidales	243	29840	2.56
Boletales	257	28914	2.48
Pleosporales	287	25927	2.23
Pezizales	268	22713	1.95
Trechisporales	241	17202	1.48
Atheliales	191	16855	1.45
Hypocreales	300	16702	1.44
Cantharellales	232	14290	1.23
Tubeufiales	172	8461	0.73
Chaetothyriales	290	8412	0.72
Xylariales	197	7327	0.63
Cystofilobasidiales	45	7050	0.61
Polyporales	135	6308	0.54
Sordariales	265	5847	0.50
unidentified	300	116894	10.04
Others	228	6202	0.53
Mortierellales	292	5187	0.45
Hymenochaetales	126	5009	0.43
Auriculariales	138	4330	0.37
Capnodiales	263	4208	0.36
Glomerales	138	4121	0.35
Chaetosphaeriales	219	3359	0.29
Archaeorhizomycetales	86	3153	0.27
Diaporthales	93	2763	0.24
Rhytismatales	110	2716	0.23
Thelebolales	158	2097	0.18
Eurotiales	224	1829	0.16
Venturiales	149	1696	0.15
Tremellales	244	1555	0.13
Trichosporonales	193	1353	0.12
Hysterangiales	12	1050	0.09
Minutisphaerales	26	723	0.06
Glomerellales	156	593	0.05
Sporidiobolales	90	531	0.05
Geoglossales	23	511	0.04
Gomphales	11	475	0.04

Table S4 (continued)

Fungal order	Taxa	Sequences	Relative abundance
Leucosporidiales	75	352	0.03
Phallales	27	350	0.03
Onygenales	53	291	0.03
Filobasidiales	59	289	0.02
Orbiliales	107	272	0.02
Ostropales	45	260	0.02
Dothideales	92	256	0.02
Mucorales	69	232	0.02
Botryosphaeriales	60	195	0.02
Holtermanniales	32	193	0.02
Myrmecridiales	41	181	0.02
Lecanorales	65	160	0.01
Saccharomycetales	45	149	0.01
Coniochaetales	62	148	0.01
Microascales	50	134	0.01
Acarosporales	5	109	0.01
Tremellodendropsidales	28	108	0.01
Verrucariales	1	76	0.01
Phacidiales	28	48	0.00
Diversisporales	14	46	0.00
Zoopagales	32	42	0.00
Olpidiales	9	36	0.00
Atractiellales	13	35	0.00
Archaeosporales	14	23	0.00
Umbelopsidales	15	18	0.00
Magnaporthales	2	16	0.00
Kriegeriales	10	15	0.00
Taphrinales	14	15	0.00
Exobasidiales	8	11	0.00
Calosphaeriales	1	10	0.00
Spizellomycetales	8	10	0.00
Myriangiales	6	9	0.00
Caliciales	2	8	0.00
Cystobasidiales	4	6	0.00
Urocystidales	4	6	0.00
Annulatascales	3	5	0.00
Boliniales	1	5	0.00
Dacrymycetales	4	5	0.00
Teloschistales	1	5	0.00
Malasseziales	3	3	0.00
Patellariales	1	3	0.00
Agaricostilbales	1	2	0.00
Entorrhizales	1	1	0.00
Ophiostomatales	1	1	0.00
Phaeomoniellales	1	1	0.00

Table S5: Classification of the top nineteen root-associated fungal orders according to functional groups. An order was classified as SYM (symbiotrophic, pink) or as SAP (saprotrophic, grey) when > 95% of the sequences of that order were classified as SYM or SAP, respectively. PAT = pathotrophic. The data set encompasses > 85% of all sequences.

Fungal order	Classification	Trophic mode	Taxa	Sequences	Relative abundance
Agaricales		PAT	9	18	0.01
Agaricales	SYM+SAP	SAP	780	121047	44.31
Agaricales		SYM	840	152130	55.69
Russulales		SAP	81	705	0.11
Russulales	SYM	SYM	867	657039	99.89
Helotiales		PAT	498	10107	4.80
Helotiales	SYM+SAP	SAP	900	136800	64.99
Helotiales		SYM	606	63576	30.20
Thelephorales		SAP	15	144	0.28
Thelephorales	SYM	SYM	768	51342	99.72
Sebacinales	SYM	SYM	663	117075	100.00
Mytilinidales	SYM	SYM	711	89424	99.93
Mytilinidiales		SAP	45	66	0.07
Boletales	SYM	SAP	45	618	1.05
Boletales		SYM	720	57990	98.95
Pleosporales		PAT	360	11271	35.74
Pleosporales	SAP+PAT	SAP	702	20241	64.18
Pleosporales		SYM	24	24	0.08
Pezizales		SAP	117	1479	2.52
Pezizales	SYM	SYM	720	57261	97.48
Trechisporales	SAP	SAP	516	38856	100.00
Atheliales		SAP	135	2127	4.25
Atheliales	SYM	SYM	528	47898	95.75
Hypocreales		PAT	846	10035	23.92
Hypocreales	SAP+PAT	SAP	894	31911	76.08
Cantharellales		PAT	9	36	0.11
Cantharellales	SYM	SAP	129	300	0.92
Cantharellales		SYM	297	32151	98.97
Tubeufiales	SAP	SAP	510	24876	100.00
Chaetothyriales		SAP	837	11373	97.16
Chaetothyriales	SAP	SYM	114	333	2.84
Xylariales		PAT	360	5382	90.02
Xylariales	SAP+PAT	SAP	255	597	9.98
Cystofilobasidiales		PAT	21	36	0.17
Cystofilobasidiales	SAP	SAP	117	21057	99.83
Polyporales	SAP	SAP	177	18108	100.00
Sordariales	SAP	SAP	750	14013	100.00

Table S6: Root-associated symbiotrophic and saprotrophic indicator taxa in the organic layer and mineral soil in three biogeographic regions. ALB = Schwäbische Alb, HAI = Hainich-Dün and SCH = Schorfheide-Chorin. Only significant fungal species belonging to the symbiotrophic (SYM) and saprotrophic (SAP) groups are shown in the table. Root-associated fungal indicator taxa with multiple or unknown trophic modes are not shown in the table. Indicator taxa for the organic layer are coloured in pink and for the mineral soil in blue.

Region	ASV ID	Orders	Genus	Species	Trophic mode	Habitat preference	Stat	*p*-value
ALB	ASV_000007	Russulales	Lactarius	*Lactarius_rubrocinctus*	SYM	Mineral	0.70	0.02
HAI	ASV_000013	Russulales	unidentified	*Russulaceae_sp.*	SYM	Mineral	0.77	0.03
HAI	ASV_000031	Sebacinales	Sebacina	*Sebacina_sp*	SYM	Mineral	0.57	0.02
SCH	ASV_000035	Mytilinidales	Cenococcum	*Cenococcum_sp*	SYM	Mineral	0.69	0.02
ALB	ASV_000040	Agaricales	Laccaria	*Laccaria_amethystina*	SYM	Organic	0.63	0.04
HAI	ASV_000040	Agaricales	Laccaria	*Laccaria_amethystina*	SYM	Organic	0.71	0.04
SCH	ASV_000040	Agaricales	Laccaria	*Laccaria_amethystina*	SYM	Organic	0.69	0.03
HAI	ASV_000044	Agaricales	Hygrophorus	*Hygrophorus_eburneus*	SYM	Mineral	0.66	0.01
SCH	ASV_000054	Russulales	Russula	*Russula_ionochlora*	SYM	Mineral	0.71	0.01
HAI	ASV_000056	Boletales	Melanogaster	*Melanogaster_sp*	SYM	Organic	0.64	0.01
SCH	ASV_000056	Boletales	Melanogaster	*Melanogaster_sp*	SYM	Organic	0.67	0.01
ALB	ASV_000062	Agaricales	Hygrophorus	*Hygrophorus_unicolor*	SYM	Mineral	0.60	0.01
HAI	ASV_000062	Agaricales	Hygrophorus	*Hygrophorus_unicolor*	SYM	Mineral	0.63	0.01
ALB	ASV_000086	Cantharellales	Membranomyces	*Membranomyces_sp*	SYM	Mineral	0.47	0.04
HAI	ASV_000089	Helotiales	Phialocephala	*Phialocephala_sp*	SYM	Organic	0.52	0.03
HAI	ASV_000106	Atheliales	Piloderma	*Piloderma_sp*	SYM	Mineral	0.81	0.01
ALB	ASV_000112	Pezizales	Tuber	*Tuber_sp*	SYM	Mineral	0.61	0.01
HAI	ASV_000112	Pezizales	Tuber	*Tuber_sp*	SYM	Mineral	0.48	0.02
ALB	ASV_000131	Russulales	unidentified	*Russulaceae_sp*	SYM	Mineral	0.68	0.01
HAI	ASV_000131	Russulales	unidentified	*Russulaceae_sp*	SYM	Mineral	0.80	0.01
SCH	ASV_000131	Russulales	unidentified	*Russulaceae_sp*	SYM	Mineral	0.50	0.02
SCH	ASV_000133	Helotiales	Acephala	*Acephala_macrosclerotiorum*	SYM	Organic	0.55	0.04
ALB	ASV_000149	Russulales	Lactarius	*Lactarius_pallidus*	SYM	Mineral	0.50	0.02

Table S6 (continued)

Region	ASV ID	Orders	Genus	Species	Trophic mode	Habitat preference	Stat	p-value
HAI	ASV_000149	Russulales	Lactarius	*Lactarius_pallidus*	SYM	Mineral	0.52	0.04
ALB	ASV_000189	Russulales	Russula	*Russula_sp*	SYM	Mineral	0.51	0.01
ALB	ASV_000237	Sebacinales	Sebacina	*Sebacina_sp*	SYM	Mineral	0.53	0.01
HAI	ASV_000243	Russulales	Lactarius	*Lactarius_sp*	SYM	Mineral	0.72	0.01
ALB	ASV_000246	Agaricales	Hymenogaster	*Hymenogaster_sp*	SYM	Mineral	0.53	0.01
ALB	ASV_000258	Agaricales	Inocybe	*Inocybe_fibrosoides*	SYM	Mineral	0.62	0.01
SCH	ASV_000261	Thelephorales	Tomentella	*Tomentella_sp*	SYM	Mineral	0.37	0.02
HAI	ASV_000271	Agaricales	Amanita	*Amanita_sp*	SYM	Mineral	0.40	0.04
SCH	ASV_000271	Agaricales	Amanita	*Amanita_sp*	SYM	Mineral	0.54	0.01
ALB	ASV_000272	Agaricales	Hygrophorus	*Hygrophorus_discoxanthus*	SYM	Mineral	0.67	0.01
ALB	ASV_000306	Russulales	Russula	*Russula_foetens*	SYM	Mineral	0.53	0.01
HAI	ASV_000306	Russulales	Russula	*Russula_foetens*	SYM	Mineral	0.35	0.03
ALB	ASV_000308	Pezizales	Otidea	*Otidea_alutacea*	SYM	Mineral	0.54	0.04
HAI	ASV_000314	Cantharellales	Clavulina	*Clavulina_sp*	SYM	Mineral	0.57	0.04
HAI	ASV_000318	Mytilinidales	Cenococcum	*Cenococcum_geophilum*	SYM	Mineral	0.64	0.01
SCH	ASV_000341	Russulales	Lactarius	*Lactarius_vellereus*	SYM	Mineral	0.46	0.03
HAI	ASV_000352	Sebacinales	Sebacina	*Sebacina_sp*	SYM	Mineral	0.67	0.01
HAI	ASV_000362	Thelephorales	Thelephora	*Thelephora_sp*	SYM	Mineral	0.60	0.01
HAI	ASV_000369	Agaricales	Hymenogaster	*Hymenogaster_sp*	SYM	Mineral	0.51	0.01
HAI	ASV_000398	Russulales	Russula	*Russula_sp*	SYM	Mineral	0.37	0.01
SCH	ASV_000398	Russulales	Russula	*Russula_sp*	SYM	Mineral	0.64	0.01
ALB	ASV_000405	Sebacinales	Sebacina	*Sebacina_sp*	SYM	Mineral	0.56	0.01
HAI	ASV_000458	Agaricales	Hygrophorus	*Hygrophorus_sp*	SYM	Mineral	0.45	0.01
ALB	ASV_000486	Pezizales	Tarzetta	*Tarzetta_sp*	SYM	Mineral	0.62	0.03
ALB	ASV_000559	Thelephorales	Tomentella	*Tomentella_sublilacina*	SYM	Organic	0.40	0.01
SCH	ASV_000559	Thelephorales	Tomentella	*Tomentella_sublilacina*	SYM	Organic	0.66	0.01
ALB	ASV_000675	Russulales	Russula	*Russula_acrifolia*	SYM	Mineral	0.58	0.01

Table S6 (continued)

Region	ASV ID	Orders	Genus	Species	Trophic mode	Habitat preference	Stat	p-value
ALB	ASV_000741	Agaricales	Hymenogaster	*Hymenogaster_sp*	SYM	Mineral	0.60	0.03
HAI	ASV_000811	Agaricales	Inocybe	*Inocybe_asterospora*	SYM	Mineral	0.62	0.01
ALB	ASV_000820	Pezizales	Tuber	*Tuber_sp*	SYM	Mineral	0.73	0.01
SCH	ASV_000823	Pezizales	Hydnotrya	*Hydnotrya_sp*	SYM	Mineral	0.81	0.01
ALB	ASV_000870	Thelephorales	Thelephora	*Thelephora_anthocephala*	SYM	Mineral	0.54	0.01
ALB	ASV_000914	Glomerales	unidentified	*Glomeraceae_sp*	SYM	Mineral	0.63	0.01
HAI	ASV_000914	Glomerales	unidentified	*Glomeraceae_sp*	SYM	Mineral	0.64	0.01
HAI	ASV_001095	Russulales	Russula	*Russula_olivacea*	SYM	Mineral	0.40	0.01
HAI	ASV_002257	Sebacinales	Sebacina	*Sebacina_sp*	SYM	Mineral	0.69	0.01
HAI	ASV_006579	Russulales	unidentified	*Russulaceae_sp*	SYM	Mineral	0.74	0.01
HAI	ASV_000041	Agaricales	Megacollybia	*Megacollybia_platyphylla*	SAP	Organic	0.61	0.01
SCH	ASV_000041	Agaricales	Megacollybia	*Megacollybia_platyphylla*	SAP	Organic	0.51	0.01
HAI	ASV_000067	Cystofilobasidiales	Mrakia	*Mrakia_aquatica*	SAP	Organic	0.66	0.01
ALB	ASV_000097	Helotiales	Lachnum	*Lachnum_sp*	SAP	Mineral	0.51	0.01
SCH	ASV_000097	Helotiales	Lachnum	*Lachnum_sp*	SAP	Mineral	0.55	0.04
ALB	ASV_000109	Agaricales	Hydropus	*Hydropus_moserianus*	SAP	Mineral	0.60	0.01
HAI	ASV_000109	Agaricales	Hydropus	*Hydropus_moserianus*	SAP	Mineral	0.35	0.03
HAI	ASV_000110	Helotiales	unidentified	*Hyaloscyphaceae_sp*	SAP	Organic	0.61	0.01
ALB	ASV_000122	Tubeufiales	Titaea	*Titaea_maxilliformis*	SAP	Organic	0.88	0.01
HAI	ASV_000122	Tubeufiales	Titaea	*Titaea_maxilliformis*	SAP	Organic	0.85	0.01
ALB	ASV_000162	Agaricales	Agrocybe	*Agrocybe_erebia*	SAP	Mineral	0.62	0.01
HAI	ASV_000162	Agaricales	Agrocybe	*Agrocybe_erebia*	SAP	Mineral	0.66	0.01
HAI	ASV_000165	Trechisporales	Luellia	*Luellia_recondita*	SAP	Organic	0.48	0.01
SCH	ASV_000165	Trechisporales	Luellia	*Luellia_recondita*	SAP	Organic	0.73	0.04
ALB	ASV_000174	Helotiales	Calycellina	*Calycellina_fagina*	SAP	Organic	0.92	0.01
HAI	ASV_000174	Helotiales	Calycellina	*Calycellina_fagina*	SAP	Organic	0.90	0.01
SCH	ASV_000174	Helotiales	Calycellina	*Calycellina_fagina*	SAP	Organic	0.88	0.01

Table S6 (continued)

Region	ASV ID	Orders	Genus	Species	Trophic mode	Habitat preference	Stat	p-value
ALB	ASV_000281	Helotiales	Encoelia	*Encoelia_sp*	SAP	Organic	0.65	0.01
ALB	ASV_000303	Trechisporales	Trechispora	*Trechispora_sp*	SAP	Organic	0.37	0.01
ALB	ASV_000384	Sordariales	Apodus	*Apodus_sp*	SAP	Organic	0.80	0.01
HAI	ASV_000384	Sordariales	Apodus	*Apodus_sp*	SAP	Organic	0.68	0.05
HAI	ASV_000401	Pleosporales	Paraconiothyrium	*Paraconiothyrium_sp*	SAP	Organic	0.51	0.01
SCH	ASV_000416	Trichosporonales	Apiotrichum	*Apiotrichum_porosum*	SAP	Organic	0.86	0.01
SCH	ASV_000589	Hypocreales	unidentified	*Bionectriaceae_sp*	SAP	Organic	0.80	0.02
HAI	ASV_000599	Helotiales	Alatospora	*Alatospora_acuminata*	SAP	Organic	0.79	0.01
ALB	ASV_000615	Hypocreales	Ijuhya	*Ijuhya_sp*	SAP	Organic	0.40	0.01
HAI	ASV_000661	Helotiales	Varicosporium	*Varicosporium_elodeae*	SAP	Mineral	0.66	0.02
ALB	ASV_000663	Hypocreales	Trichoderma	*Trichoderma_polysporum*	SAP	Organic	0.79	0.02
SCH	ASV_000677	Helotiales	unidentified	*Hyaloscyphaceae_sp*	SAP	Mineral	0.69	0.02
ALB	ASV_000772	Chaetothyriales	Cladophialophora	*Cladophialophora_sp*	SAP	Mineral	0.86	0.01
HAI	ASV_000774	Agaricales	Pseudoclitocybe	*Pseudoclitocybe_cyathiformis*	SAP	Organic	0.37	0.03
SCH	ASV_000850	Sordariales	Cephalotheca	*Cephalotheca_sp*	SAP	Organic	0.66	0.03
HAI	ASV_000955	Agaricales	Lycoperdon	*Lycoperdon_nigrescens*	SAP	Organic	0.52	0.02
SCH	ASV_001094	Helotiales	Cryptosporiopsis	*Cryptosporiopsis_sp*	SAP	Organic	0.64	0.01
SCH	ASV_001166	Helotiales	unidentified	*Hyaloscyphaceae_sp*	SAP	Organic	0.73	0.01
ALB	ASV_002043	Chaetothyriales	Cladophialophora	*Cladophialophora_sp*	SAP	Organic	0.78	0.01
HAI	ASV_002043	Chaetothyriales	Cladophialophora	*Cladophialophora_sp*	SAP	Organic	0.79	0.01
SCH	ASV_002658	Archaeorhizomycetales	Archaeorhizomyces	*Archaeorhizomyces_sp*	SAP	Organic	0.57	0.02
HAI	ASV_003876	Agaricales	Agrocybe	*Agrocybe_erebia*	SAP	Mineral	0.49	0.01
ALB	ASV_003921	Pleosporales	Subplenodomus	*Subplenodomus_galicola*	SAP	Organic	0.58	0.01
ALB	ASV_004119	Atheliales	Athelopsis	*Athelopsis_lembospora*	SAP	Mineral	0.66	0.01
ALB	ASV_007068	Agaricales	Cyclocybe	*Cyclocybe_sp*	SAP	Mineral	0.53	0.01
